# Skeletal myosin binding protein-C isoforms regulate thin filament activity in a Ca^2+^-dependent manner

**DOI:** 10.1038/s41598-018-21053-1

**Published:** 2018-02-08

**Authors:** Brian Leei Lin, Amy Li, Ji Young Mun, Michael J. Previs, Samantha Beck Previs, Stuart G. Campbell, Cristobal G. dos Remedios, Pieter de P. Tombe, Roger Craig, David M. Warshaw, Sakthivel Sadayappan

**Affiliations:** 1 Department of Cell and Molecular Physiology, Health Sciences Division, Loyola University Chicago, Maywood, IL 60153 USA; 2 Department of Molecular Physiology and Biophysics, University of Vermont, Burlington, VT 05405 USA; 3 Bosch Institute, Discipline of Anatomy and Histology, University of Sydney, Sydney, 2006 Australia; 4 Department of Cell and Developmental Biology, University of Massachusetts Medical School, Worcester, MA 01655 USA; 5 Departments of Biomedical Engineering and Cellular and Molecular Physiology, Yale University, New Haven, CT 06520 USA; 6 Department of Structure and Function of Neural Network, Korea Brain Research Institute, Dong-gu, Daegu, Korea

## Abstract

Muscle contraction, which is initiated by Ca^2+^, results in precise sliding of myosin-based thick and actin-based thin filament contractile proteins. The interactions between myosin and actin are finely tuned by three isoforms of myosin binding protein-C (MyBP-C): slow-skeletal, fast-skeletal, and cardiac (ssMyBP-C, fsMyBP-C and cMyBP-C, respectively), each with distinct N-terminal regulatory regions. The skeletal MyBP-C isoforms are conditionally coexpressed in cardiac muscle, but little is known about their function. Therefore, to characterize the functional differences and regulatory mechanisms among these three isoforms, we expressed recombinant N-terminal fragments and examined their effect on contractile properties in biophysical assays. Addition of the fragments to *in vitro* motility assays demonstrated that ssMyBP-C and cMyBP-C activate thin filament sliding at low Ca^2+^. Corresponding 3D electron microscopy reconstructions of native thin filaments suggest that graded shifts of tropomyosin on actin are responsible for this activation (cardiac > slow-skeletal > fast-skeletal). Conversely, at higher Ca^2+^, addition of fsMyBP-C and cMyBP-C fragments reduced sliding velocities in the *in vitro* motility assays and increased force production in cardiac muscle fibers. We conclude that due to the high frequency of Ca^2+^ cycling in cardiac muscle, cardiac MyBP-C may play dual roles at both low and high Ca^2+^. However, skeletal MyBP-C isoforms may be tuned to meet the needs of specific skeletal muscles.

## Introduction

Myosin binding protein-C (MyBP-C) is a striated muscle protein that regulates contraction and consists of three isoforms known as slow-skeletal, fast-skeletal, and cardiac (ssMyBP-C, fsMyBP-C, and cMyBP-C), encoded by *MYBPC1*, *MYBPC2* and *MYBPC3*, respectively^1–3^. As their names would suggest, ssMyBP-C and fsMyBP-C are predominantly expressed in adult skeletal muscle tissue, while cMyBP-C is the predominant isoform in the heart^4^. However, the naming convention belies the complex expression of MyBP-C isoforms, as studies have demonstrated that ssMyBP-C is expressed at low levels in the mammalian atrium^5^, and fsMyBP-C is present in the heart in murine models of heart failure^6^. Few studies exist on ssMyBP-C^4^, with fewer still on fsMyBP-C^7^. As such, their individual contributions to contractile regulation are essentially unknown. The clinical significance of understanding the function of each isoform is clear: mutations in cMyBP-C are the most common cause of hypertrophic cardiomyopathy (HCM)^8^, while more recently, mutations in ssMyBP-C and fsMyBP-C that cause skeletal muscle myopathies, such as distal arthrogryposis, have been discovered^9–11^.

cMyBP-C is the best-characterized isoform, and its N-terminal region regulates contractility through its interaction with both myosin subfragment 2 (S2) and actin^12–14^. Myosin and actin are the major thick and thin filament proteins that interact to produce filament sliding at the molecular level. Interestingly, the cMyBP-C N-terminal regulatory region activates the thin filament by shifting the position of tropomyosin (Tm) from a “blocked” position to a “closed” position^13^, exposing myosin-binding sites on F-actin. Shifting Tm is the same mechanism by which Ca^2+^ and the troponin complex (Tn) activate the thin filament to enable contraction^15^. However, Tm shift by Tn and MyBP-C are not redundant mechanisms. The centralized location of MyBP-C within the sarcomere appears critical for the correction of an inhomogeneity in activation resulting from a spatiotemporal Ca^2+^ gradient^16^. What seems redundant is the presence of three distinct MyBP-C isoforms that are conditionally co-expressed. However, each MyBP-C isoform is structurally unique, and as our data show, functionally unique.

**Figure 1:**
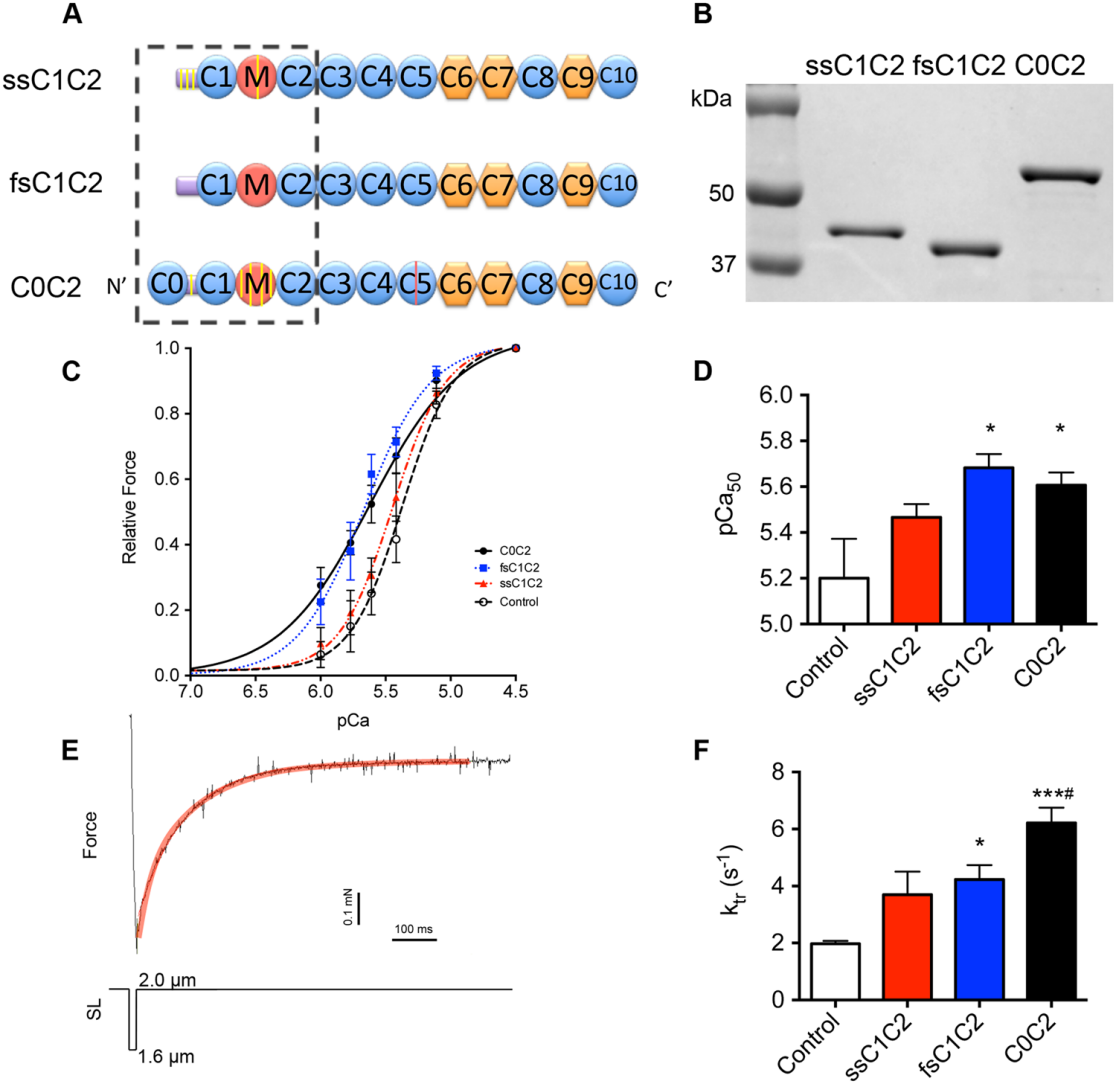
Regulation of muscle contraction by MyBP-C isoforms was determined using recombinant proteins representing the N-terminal region of slow-skeletal, fast-skeletal, and cardiac isoforms of MyBP-C. (**A**) Schematic diagram of full-length slow-skeletal, fast-skeletal, and cardiac MyBP-C isoforms (ssMyBP-C, fsMyBP-C, and cMyBP-C, respectively). Domains are numbered C0 to C10 from the N-terminus, and proline-alanine region (PA) is common to all isoforms. Circles denote immunoglobulin-like domains, and pentagons represent fibronectin type 3 domains. Yellow lines identify known phosphorylation sites; red lines indicate cardiac-specific insert in C5 domain of cMyBP-C. (**B**) SDS-PAGE demonstrates the relative size and purity of each MyBP-C recombinant protein, encompassing the N-terminal region, up to and including the C2 domain. (**C**–**F**) Permeabilized ventricular rat papillary muscles were left untreated (control, white column) or were incubated with 10 µM ssC1C2 (red), fsC1C2 (blue), and C0C2 (black) and allowed to undergo muscle contraction analysis by the Force-ATPase assay. (**C**) Relative force-pCa curves and (**D**) quantification of pCa_50_ values from the relative force-pCa curves demonstrate significant increases of fsC1C2 and C0C2 in Ca^2+^-sensitivity of force development. (**E**) Rate of tension redevelopment (*k*_tr_) was determined using a rapid release and restretch maneuver at maximal Ca^2+^ levels (pCa 4.5) and submaximal Ca^2+^ levels (pCa 6). Top trace is of (**F**) at pCa 6, and *k*_tr_ was significantly enhanced by fsC1C2 and C0C2. Graphs represented as mean ± SEM, *p < 0.05 *vs*. controls, **p < 0.01 *vs*. controls, ^#^p < 0.05 *vs*. ssC1C2/fsC1C2, n = 7–9 animals.

All MyBP-C isoforms consist of a linear array of seven immunoglobulin-like and three fibronectin-like domains numbered C1 to C10, as well as unique N-terminal structures: an M-domain between C1 and C2^17^ and a proline/alanine-rich (PA) sequence preceding the C1 domain (Fig. 1A). Critically important differences exist within the N-terminal region up to the C2 domain (Fig. 1A, dotted box). For example, cMyBP-C has a cardiac-specific IgG domain (C0) at its N-terminus that binds myosin regulatory light chain (MLC2) and actin *in vitro*^18^. cMyBP-C also has four phosphorylation sites in its M domain^19^ and one more in its PA region^20^. In contrast, the ssMyBP-C isoform has only one phosphorylation site in its M domain and three in its PA region^21^ and no known phosphorylation sites have been found in fsMyBP-C. Sequence comparisons within this region suggest unique N-terminal structural differences (Supplemental Table S1), prompting us to determine the impact of the three major MyBP-C isoforms on muscle contractility, with a focus on heart muscle.

Our objective is to parse out the differences among the N-terminal regulatory regions (up to and including the C2 domain) of each individual MyBP-C isoform (Fig. 1A and Supplemental Table S1). How these isoforms vary in their interactions with the thin filament may explain why three distinct isoforms are expressed, and the answers may lay the groundwork for future studies in the skeletal MyBP-C field. Thus, we generated recombinant N-terminal peptides of ssMyBP-C, fsMyBP-C and cMyBP-C (ssC1C2, fsC1C2, and C0C2, respectively) and characterized their functions using a wide combination of biophysical techniques. We used three-dimensional reconstructions of electron microscopy images, measurements of fiber force-ATPase activity, *in vitro* motility assays, cardiomyocyte unloaded shortening, and computer simulation approaches to determine how these N-terminal fragments affect muscle function. Overall, our studies demonstrate that ssMyBP-C and cMyBP-C share similar regulatory function at low Ca^2+^. Conversely, fsMyBP-C and cMyBP-C share similar regulatory function at higher Ca^2+^. Steady-state functional experiments demonstrate that all MyBP-C isoforms sensitize the thin filaments to Ca^2+^ and that the extent of sensitization is isoform-specific. Structural studies demonstrate that each isoform affects tropomyosin position on F-actin, which, in turn, may regulate function in a graded manner. Lastly, dynamic functional experiments, which measured *in vitro* and *in silico* unloaded myocyte shortening, confirmed our principal expectation^22^: that the greater capacity to activate the thin filament results in slower relaxation kinetics. Our results suggest that cMyBP-C has distinct regulatory functions over a full range of Ca^2+^, possibly because of the dynamic range of intracellular Ca^2+^ experienced on a beat-to-beat basis in the heart. Conversely, differential expression of ssMyBP-C and fsMyBP-C in skeletal muscles may have evolved to fine-tune the role of skeletal MyBP-C in fast-twitch and slow-twitch muscles. The relatively small amounts of ssMyBP-C and fsMyBP-C expressed in the heart may also subtly augment cardiac muscle function. Taken together, these data are the first to reveal differences in the underlying molecular and cellular regulatory functionality of all three MyBP-C N-termini.

## Results

### cMyBP-C and fsMyBP-C N-terminal fragments activate force development at higher Ca^2+^levels

cMyBP-C is known to activate the thin filament^13,16,23^ and thus promote Ca^2+^-sensitivity and submaximal force development^24^ (Supplemental Figure S1D). To determine whether ssMyBP-C and fsMyBP-C also affect force development at the muscle fiber level, we applied N-terminal MyBP-C fragments to a force-ATPase assay from pCa 6–4.5. The force-ATPase assay combines isometric force measurements with Ca^2+^ (pCa), myosin ATPase activity, and stiffness relationships (Fig. 1 and Supplemental Figure S1E and F). In this assay, permeabilized rat papillary muscle fibers were attached to aluminum t-clips. Each fiber bundle was then hung between a force transducer and a length-controller, followed by incubation with vehicle control or one of three experimental groups: 10 μM ssC1C2, fsC1C2, or C0C2. Unlike permeabilized myocytes, these thick bundles of papillary muscle fibers necessitated the use of high concentrations (10 µM) of fragments because of the higher myosin concentration. The presence of 10 µM exogenous N-terminal fragments had no significant differential effects on force or fiber stiffness at maximal Ca^2+^ levels (pCa 4.5) (Supplemental Figure S1D and E). Interestingly, exogenous C0C2 and fsC1C2 both increased Ca^2+^-sensitivity of force development (Fig. 1C and D), and ssC1C2 appeared to trend towards increasing Ca^2+^ sensitivity within the Ca^2+^ range tested. C0C2 and fsC1C2 increase in Ca^2+^-sensitivity also resulted in increased submaximal force generation (Supplemental Figure S1D), suggesting that these isoforms regulate some aspect of myosin-actin interaction.

To determine the mechanism by which each isoform increased Ca^2+^-sensitivity, we analyzed the rate of tension redevelopment (*k*_tr_), which is an estimation of both thin filament activation and cross-bridge cycling kinetics (Fig. 1E). To measure k_tr_, we applied a rapid release-and-restretch maneuver of the muscle fiber. During this maneuver, cross-bridges within the sarcomere are mechanically detached by rapidly shortening and immediately restretching the muscle fibers to their initial resting sarcomere length. Tension is allowed to redevelop in the presence of N-terminal fragments, and the timecourse of the fit of the tension redevelopment curve estimates thin filament activation and cross-bridge cycling kinetics. These experiments were conducted at submaximal pCa 6 (Fig. 1F and Supplemental Figure S1G) and maximal pCa 4.5 (Supplemental Figure S1H) calcium levels to determine the effects of each fragment on *k*_tr_ at submaximal and maximal activation levels. While no change was observed in *k*_tr_ at maximal activation (pCa 4.5), we show that fsC1C2 and C0C2 increased *k*_tr_ relative to controls at submaximal activation levels (pCa 6) (Fig. 1F), suggesting that these fragments promote thin filament activation and/or cross-bridge cycling to regulate Ca^2+^-sensitivity (Fig. 1D). This data correlates with an increase in submaximal force observed when fibers were incubated with fsC1C2 and C0C2 (Supplemental Figure S1D). While ssC1C2 trended towards increased *k*_tr_ (Fig. 1D and F), its effect was not statistically significant from that of controls, suggesting that ssC1C2 may not regulate contraction at all within this range (pCa 6–4.5) and that the roles of different MyBP-C isoforms are Ca^2+^-dependent. This result called for functional assays with increased sensitivity to determine if MyBP-C regulates contraction at even lower Ca^2+^ levels. Therefore, we utilized *in vitro* motility assays to probe MyBP-C function from pCa 9 to pCa 4.

### MyBP-C N-terminal fragments differentially activate and inhibit thin filament motility in a Ca^2+^-dependent manner

**Figure 2:**
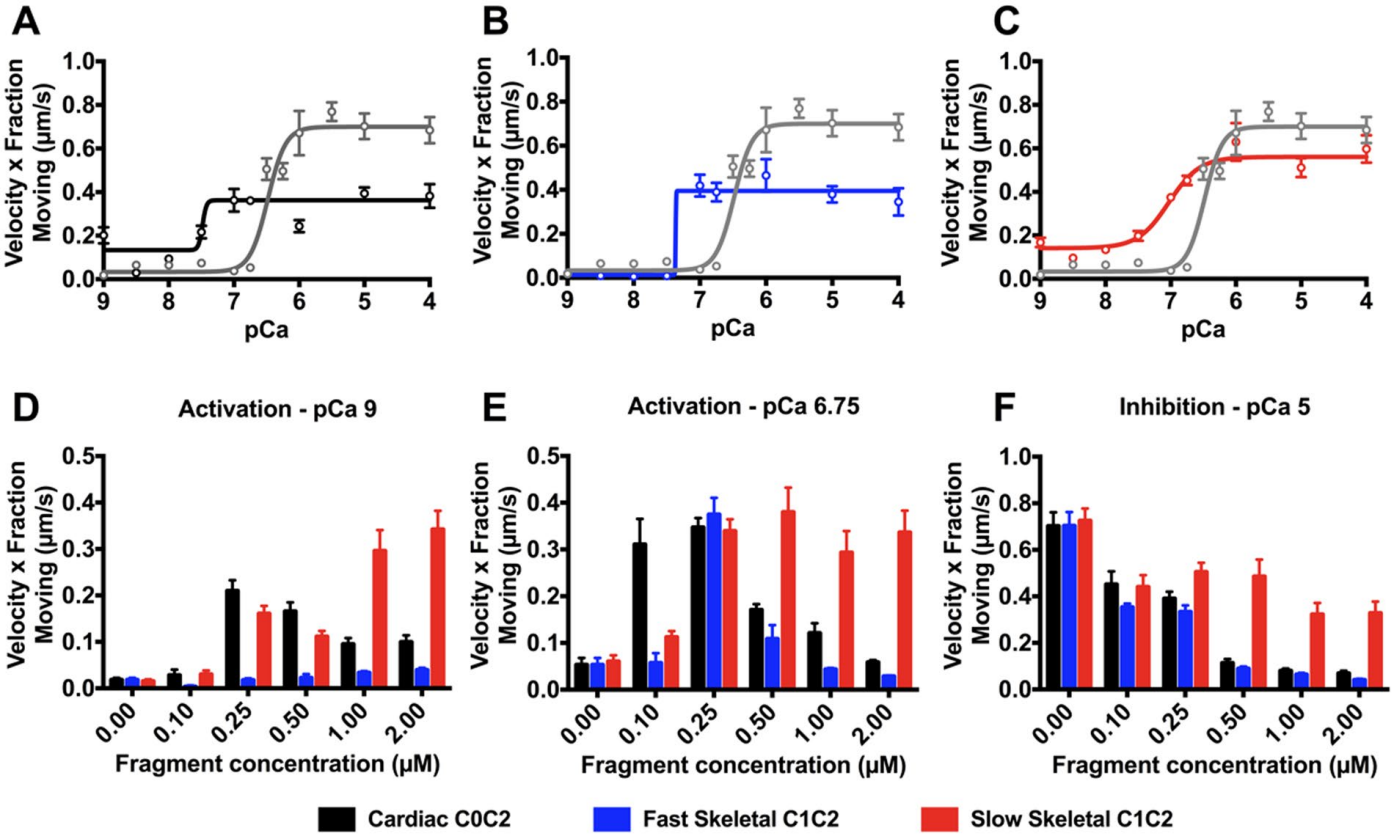
*In vitro* motility assays demonstrate MyBP-C regulation by promoting and inhibiting motility of native thin filaments (NTFs) at low and high Ca^2+^, respectively. (**A**–**C**) Control NTFs (grey line) show a sigmoidal increase in activation in response to increasing calcium levels. C0C2, fsC1C2, and ssC1C2 (0.25 µM) all shift this response to the left, indicating an increased activation effect at lower Ca^2+^ levels. (**A**) C0C2 activates NTF sliding velocities at both low Ca^2+^ levels and inhibits NTF motility at high Ca^2+^ levels. (**B**) fsC1C2 is unable to activate NTF motility at low Ca^2+^ levels, but inhibits NTF motility at higher Ca^2+^ levels. (**C**) Conversely, ssC1C2 activates NTF motility at low Ca^2+^ levels, but lacks the capacity to limit NTF motility at high Ca^2+^ levels. Effects of MyBP-C N-termini varied, depending on concentration, as demonstrated by dose-dependent responses of C0C2, fsC1C2, and ssC1C2 on NTF motility at (**D**) pCa 9 (**E**) pCa 6.75 and (**F**) pCa 5. Graphs represented as mean ± SEM.

The N-terminal region of cMyBP-C sensitizes thin filaments to Ca^2+^ ^13^, but it slows thin filament sliding velocities when the thin filament is fully activated^14^. To investigate how skeletal MyBP-C isoforms modulate contraction at low Ca^2+^ levels compared to maximal Ca^2+^ levels, we applied our N-terminal fragments to *in vitro* motility assays. The *in vitro* motility assay was used to characterize the Ca^2+^-dependent motion of mouse cardiac native thin filaments (NTF) over a bed of mouse ventricular myosin in the presence of ssC1C2, fsC1C2, and C0C2 fragments (Fig. 2). Thin filament motion was measured as the product of the fraction of moving filaments and their sliding velocities, which is an estimate of Ca^2+^-dependent thin filament activation and actomyosin contractility (Supplemental Figure S2). By plotting velocity x fraction of moving filaments, we were able to evaluate the impact of the N-terminal fragments (0.25 µM) on both thin filament activation at low calcium levels and actomyosin contractility once fully calcium activated. The concentration of monomeric myosin incubated in each flow cell was 100 µg/ml, or 217 nM^25^. Addition of 0.25 µM MyBP-C fragments allowed for examination of the effects at an approximate 1:1 ratio to myosin. As expected, C0C2 sensitized native thin filament motility at low calcium concentrations (pCa 9–7) and slowed thin filament velocities by 43% at high calcium, pCa 4. At the same 0.25 µM fragment concentration, both fsC1C2 and ssC1C2 sensitized native thin filaments to calcium with ssC1C2, showing activation potency equal to that of C0C2 at pCa 9–7 (Fig. 2A and D). Indeed, this demonstrates that ssC1C2 does regulate contraction, albeit at very low Ca^2+^ levels. In contrast, fsC1C2 had only minimal activation capacity at low Ca^2+^ (pCa 7), whereas fsC1C2 mainly acted to inhibit NTF velocities to the degree equal to that of C0C2 (Fig. 2B and E). In stark contrast, the ssC1C2 fragment only modestly regulated NTF sliding at high Ca^2+^ levels (Fig. 2C and F). These differences were observed over a range of fragment concentrations (0–2.0 μM) (Fig. 2D–F) probed in a relaxed state at pCa 9, at the cusp of physiological activation at pCa 6.75, and at maximal activation at pCa 4. These results clearly demonstrate the calcium-dependent manner in which each isoform regulates motility. For example, at pCa 9, fsC1C2 shows little capacity to activate NTFs across all fragment concentrations when compared to C0C2 and ssC1C2. At the other extreme, ssC1C2 was not as effective in inhibiting NTF sliding velocity at any pCa value tested (Fig. 2F). The motility data demonstrate that each fragment may have evolved to operate over niche ranges of Ca^2+^ to match their respective repertoire of contractile regulation. To illustrate this point, ssC1C2 and C0C2 activate NTF sliding velocities at pCa 9-7, while fsC1C2 and C0C2 inhibit NTF sliding velocities at pCa 6-4. These functional results suggest that MyBP-C isoforms share similarities in regulatory function. Furthermore, these similarities depend on the [Ca^2+^] and are thus associated with distinct stages of contraction.

### MyBP-C N-termini activate thin filaments by binding to F-actin and shifting tropomyosin

**Figure 3:**
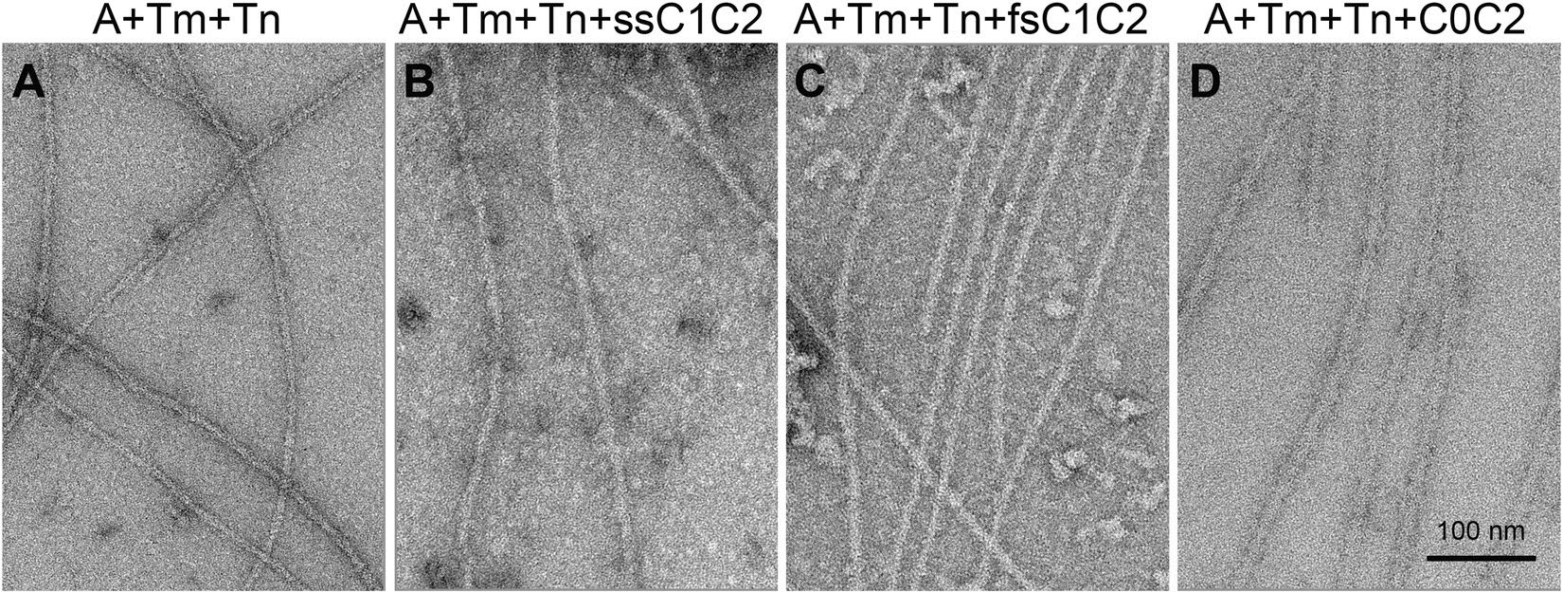
Electron microscopy (EM) shows binding of ssC1C2, fsC1C2, and C0C2 to reconstituted thin filaments. (**A**) Control thin filaments reconstituted from F-actin, tropomyosin, and troponin at a molar ratio of 7:2:2 in low Ca^2+^ buffer and imaged by negative staining. (**B**–**D**) Reconstituted thin filaments decorated with (**B**) ssC1C2 (**C**) fsC1C2 and (**D**) C0C2. Binding of these N-terminal fragments is evident both from the images and from an increase in filament diameter (Supplemental Table S3).

We have previously shown that binding of cMyBP-C N-terminal fragments (C0C2, C0C3) to thin filaments, in the absence of Ca^2+^, shifts tropomyosin to the “closed” position, which favors weak cross-bridge formation^13^. We hypothesized that binding of the skeletal MyBP-C isoforms to thin filaments might have a similar impact. To test this hypothesis, reconstituted thin filaments (composed of actin (A), tropomyosin (Tm), and the troponin (Tn) complex) were decorated with MyBP-C N-terminal fragments (ssC1C2, fsC1C2 and C0C2) at low Ca^2+^ and imaged by negative staining EM (Fig. 3A–D). An increase in filament diameter was observed in the presence of each MyBP-C N-terminal fragment, demonstrating the ability of each one to bind to the thin filament (Fig. 3A–D, Supplemental Table S3).

**Figure 4:**
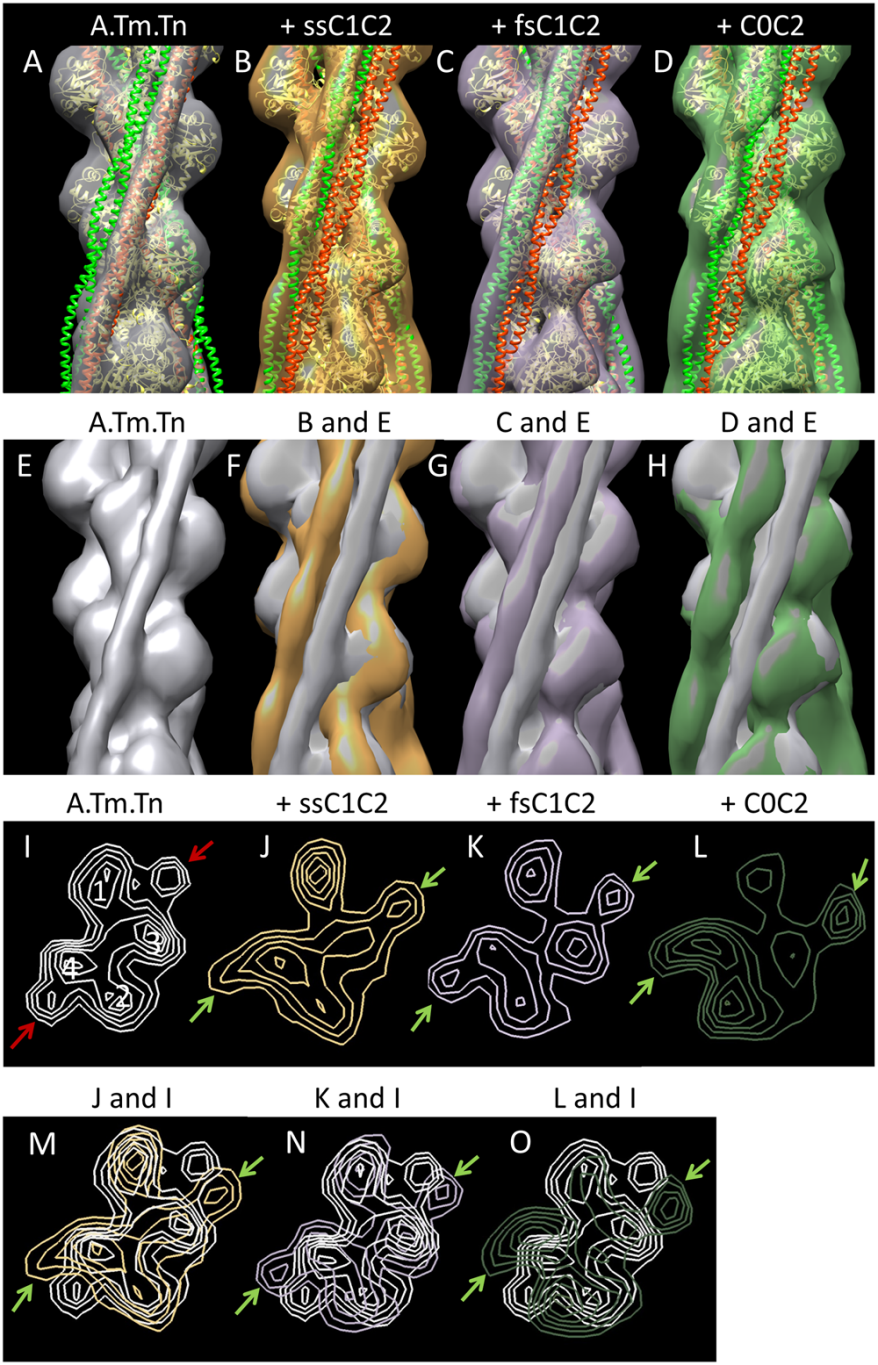
3D reconstruction of thin filaments demonstrates MyBP-C N-termini shift of tropomyosin in the absence of Ca^2+^. (**A**) Reconstruction of reconstituted thin filament (F-actin, tropomyosin, troponin). Actin atomic structure (yellow ribbon) has been fitted into the reconstruction (grey envelope). Red and green helices represent Tm in the known blocked and closed positions on the thin filament, respectively. In this control filament, at low Ca^2+^, Tm occupies the blocked position (grey cylinder enclosing red Tm helix). Tn is averaged out as it does not follow the helical symmetry of actin used to carry out the reconstructions. The addition of (**B**) ssC1C2, (**C**) fsC1C2, and (**D**) C0C2 causes a shift in Tm azimuth towards the closed position, with C0C2 causing the largest shift and fsC1C2 the smallest. These variable shifts are further revealed when each decorated reconstruction is superimposed on the actin:tropomyosin:troponin control, both in surface view (**E**–**H**) and in cross-sectional views of the reconstructions (**I**–**O**) (*cf*. ref.^14^). Actin subdomains 1–4 are marked in **I**. Red arrows indicate Tm in blocked position in low Ca^2+^ control filament; green arrows show shifted position of Tm in low Ca^2+^ decorated filaments.

Next, 3D reconstructions were computed from these images to visualize whether binding of the different MyBP-C isoforms caused any shift in tropomyosin position (Fig. 4). To assess this, the positions of tropomyosin in the known blocked and closed positions were superimposed on the reconstructions (red and green helices, respectively; Fig. 4A–D). Results showed that all three MyBP-C fragments had shifted tropomyosin from the blocked position towards the closed position, as induced by Ca^2+^ ^13,26^. C0C2 had the greatest effect, moving Tm significantly beyond the closed position (Fig. 4D). fsC1C2 shifted Tm as far as the closed position, while ssC1C2 was in between (Fig. 4B,C). These differences could be clearly illustrated when reconstructions of each of the MyBP-C-decorated filaments were superimposed on the low Ca^2+^ control filament and viewed either in surface view (Fig. 4E–H) or as cross sections (Fig. 4I–O). The greater ability of C0C2 and ssC1C2 to displace Tm suggests that they would be more potent activators of thin filaments at low Ca^2+^ levels than fsC1C2, underscoring the Ca^2+^-dependent manner in which MyBP-C isoforms differentially regulate contraction. This trend is consistent with motility data that demonstrated ssC1C2 and C0C2 activate filament sliding at low (pCa 9) Ca^2+^ levels, while fsC1C2 only activated sliding at intermediate Ca^2+^ levels (pCa 7) (Fig. 2). The different effects of each isoform on Tm shift strongly suggest that thin filament activation is one mechanism by which MyBP-C isoforms regulate contraction at low Ca^2+^.

### Steady-state thin filament activation may translate to prolonged dynamic relaxation kinetics

**Figure 5:**
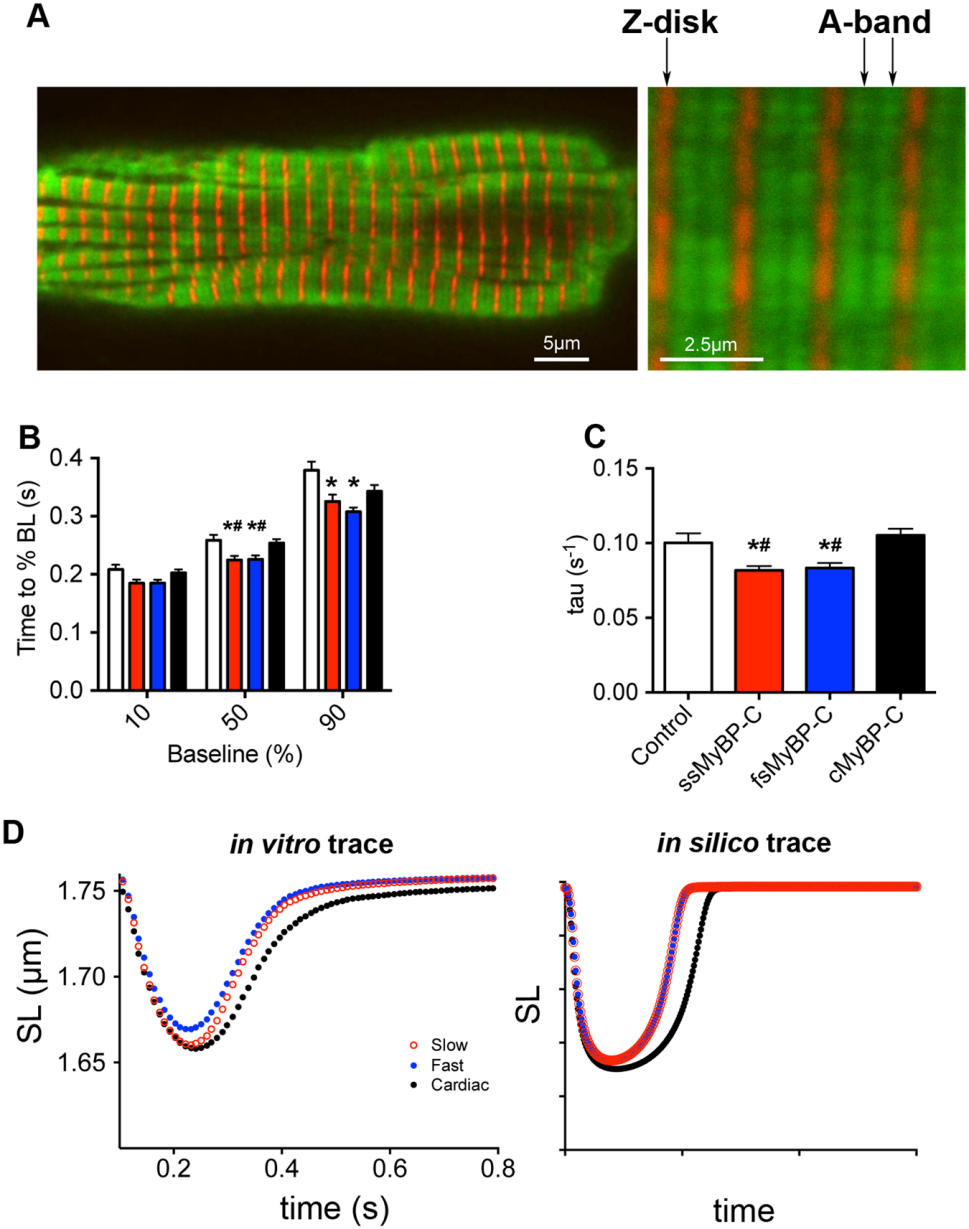
Greater activation of thin filament results in prolonged diastole. (**A**) To determine how each isoform regulates dynamic contraction, full-length MyBP-C adult rat ventricular myocytes (ARVM) were infected with adenoviral constructs (MOI 1000) overexpressing full-length, cMyc-tagged slow-skeletal, fast-skeletal, or cardiac MyBP-C, followed by 48 h culture. (**B**) Immunofluorescence (IF) imaging demonstrates localization of adenoviral-mediated expression of MyBP-C isoforms (green) within the sarcomere, as delineated by α-actinin (red). (**C**,**D**) Unloaded shortening was measured by changes in sarcomere length (SL) during dynamic contraction and relaxation (ARVM paced at 1 Hz, 20 V, 2 ms). (**C**) Relaxation kinetics was measured by time to % baseline, how fast the cell returns to 10, 50, and 90% of resting SL, and (**D**) relaxation constant tau, a logarithmic fit of the relaxation curve. (**E**) Changes in relaxation kinetics were evident by combined traces, and *in silico* simulations demonstrate that greater thin filament activation can contribute to relaxation kinetics. Graphs represented as mean ± SEM, *p < 0.05 *vs*. uninfected controls, ^#^p < 0.05 *vs*. cMyBP-C.

Previously, thin filament activation by MyBP-C was postulated to prolong relaxation kinetics by promoting the duration of attached cross-bridges^22^. The differential thin filament activation capacity of each MyBP-C isoform regulates contraction, but may also impact relaxation. Unloaded cardiomyocyte mechanics were utilized to provide contraction-relaxation velocities. These are readouts of actomyosin contractility and, hence, a direct comparison with the *in vitro* motility studies. The velocity:pCa results from both model systems can be used to characterize changes in Ca^2+^ sensitivity as a result of the MyBP-C fragments and suggest potential changes in relaxation potential. Therefore, we hypothesized that the observed changes in steady-state regulation by MyBP-C isoforms allow MyBP-C to regulate dynamic relaxation kinetics, as well. To test this hypothesis, our steady-state experiments examined MyBP-C isoform-specific modulation of force-pCa in permeabilized fibers and thin filament activation/inhibition of NTF motility (Figs 1–4, Table 1). To test if MyBP-C isoforms could alter relaxation kinetics during dynamic contraction, we utilized intact, isolated cardiomyocytes, and they were allowed to freely contract and relax. We analyzed unloaded shortening kinetics of electrically stimulated (2.0 ms, 1 Hz, 20 V) adult rat ventricular myocytes (ARVM) infected with adenoviral constructs overexpressing full-length, Myc-tagged ssMyBP-C, fsMyBP-C, or cMyBP-C in culture for 48 hours. Full-length MyBP-C isoforms were used because abnormal localization and function of N-terminal fragments would result from the absence of the C-terminal region^27^, even though the N-terminal region localizes to the A-band^24^. Immunofluorescence imaging revealed that adenoviral-mediated MyBP-C isoforms expressed and localized properly to the sarcomere A-band where myosin thick filaments reside (Fig. 5A and Supplemental Figure S3). Densitometry analysis of Western blots also revealed partial replacement of endogenous cMyBP-C with adenoviral-mediated constructs (Supplemental Figure S4A and B). Unloaded shortening experiments demonstrated that cMyBP-C prolonged relaxation kinetics relative to ssMyBP-C and fsMyBP-C, as determined by time to percent baseline, i.e., how fast the cell returned to resting sarcomere length (SL) and tau, which is the relaxation constant based on a fit of the relaxation trace (Fig. 5B and C). In combination with our steady-state observations, greater thin filament activation has been suggested to prolong relaxation^22^, which explains the effect cMyBP-C had on our ARVM system.

**Table 1:** Summary of steady-state experimental results.

Experimental Assay	System	Concentration of N’ fragment	Results at lower Ca^2+^ (pCa 9–7)	Results at higher Ca^2+^ (pCa 6–4.5)
Force-ATPase	Adult Rat Papillary Muscle Fiber Bundles	10 µM	N/A	C0C2 and fsC1C2 increased force, Ca^2+^ sensitivity, k_tr_
*In vitro* Motility Assays	Mouse myosin and Native Thin Filaments	0.25 µM 0.10–2.0 µM	C0C2 and ssC1C2 activate NTF motility	C0C2 and fsC1C2 inhibit NTF motility
3D reconstruction of EM images	Reconstituted thin filament	6 µM	Graded shifts in tropomyosin position (C0C2 > ssC1C2 > fsC1C2)	N/A

The table summarizes all steady-state experiments performed in the present studies, experimental systems and concentration of the MyBP-C fragments used. Results are summarized at lower and higher Ca^2+^ concentration.

To determine whether the greater capacity of cMyBP-C to activate the thin filament also explains its regulation of relaxation kinetics during contraction, we utilized an online muscle simulator (OMS) that simulates unloaded shortening of a single cardiomyocyte^28^. By adjusting parameters that accounted solely for differential capacity to activate the thin filament (k_on_), we could use the OMS for specific modelling of steady-state parameters that affect unloaded shortening (Fig. 5D and Supplemental Table S4). The resulting *in silico* unloaded shortening traces recapitulated *in vitro* composite traces and data (Fig. 5B–D), suggesting that greater thin filament activation by cMyBP-C regulates relaxation. Collectively, these data demonstrate that regulation of steady-state contraction by different MyBP-C isoforms can contribute to relaxation kinetics in dynamic contraction.

## Discussion

Contractile proteins often have cardiac and skeletal isoforms^29^, and MyBP-C is no exception. Unique expression profiles demonstrate that these isoforms can be co-expressed in health and disease^5,6^, underscoring the need to determine functional differences and similarities among all three MyBP-C isoforms. Additionally, as new disease-causing mutations in the two skeletal MyBP-C isoforms are discovered^9–11^, studies that provide insight into the function of ssMyBP-C and fsMyBP-C are becoming increasingly relevant^7,30^. Indeed, our results do demonstrate clear differential functional roles for ssMyBP-C, fsMyBP-C, and cMyBP-C in regulating muscle contraction.

One key finding was that MyBP-C isoforms activate the thin filament within functional Ca^2+^ niches. Here, we demonstrate that ssMyBP-C promotes NTF sliding at low [Ca^2+^], and fsMyBP-C inhibits NTF sliding at high [Ca^2+^], while cMyBP-C has the capacity to regulate both at both low and high calcium. Furthermore, the capacity of each MyBP-C isoform to regulate within a particular range of [Ca^2+^] is dependent on the ability of each to bind to, and activate the thin filament. At low [Ca^2+^], ssC1C2 and C0C2 shifted Tm beyond the ‘closed’ position, which would be expected to activate the thin filament by partially exposing the myosin-binding site on actin (Fig. 4F–H)^13^. This increased ability to activate the thin filament correlated with increased NTF sliding velocities at low [Ca^2+^] (pCa 9), but had no effect at high [Ca^2+^]. Thus, MyBP-C appears to have a key role in initiating contraction. Conversely, fsC1C2 activation of sliding did not occur until intermediate Ca^2+^ levels (pCa 7). Where fsC1C2 exhibited marked regulation was its ability to reduce NTF sliding velocities at high [Ca^2+^] to a degree equal to that of C0C2. Correspondingly, fiber studies demonstrated that fsC1C2 and C0C2 enhanced force production above pCa 6. In contrast, ssC1C2 did not appear to have a significant regulatory function in the fiber experiments, possibly because the lower Ca^2+^ limit of this assay was pCa 6, and the regulatory capacity of ssC1C2 is limited to lower [Ca^2+^]. Interestingly, when we compared the results of the fiber and motility assays, we concluded that fsC1C2 and C0C2 share similarities in contractile regulation at intermediate and high Ca^2+^ levels (Supplemental Figure S1). These isoforms may promote force generation by contributing to the stability and maintenance of strongly bound cross-bridges^31^.

It is important to note that the present studies focus on MyBP-C N-terminal interactions with the thin filament and do not preclude regulation by MyBP-C through its interactions with myosin. Indeed, MyBP-C may form a “C-bridge,” in which full-length MyBP-C binding to the thin filament creates a secondary thick-to-thin filament interaction, the functional consequences of which are not well understood. This interaction may allow MyBP-C to interact with both actin and the myosin head region. The MyBP-C/actin interaction promotes the “on” state of the thin filament (f_app_), competing with myosin for an actin-binding site specifically within the C-zone and/or placing a load on actin to limit contractility^32,33^. Alternatively, MyBP-C may place a load on the myosin head region, limiting cross-bridge formation by maintaining myosin in its off state (g_app_)^19^. Whether MyBP-C binds to the thin or thick filament may depend on the stage of contraction and, by association, the levels of Ca^2+^ in the sarcomere. Recent studies have highlighted Ca^2+^-dependent regulation by cMyBP-C^34,35^ and the physiological role for MyBP-C in preventing muscle hypercontractility^36^.

A second key finding was that all MyBP-C isoforms alter thin filament properties to regulate Tm shift on actin. Both the binding affinity of MyBP-C to actin and its effects on the thin filament underlie the ability of MyBP-C to regulate contraction at various ranges of Ca^2+^, suggesting, in turn, that MyBP-C may both inhibit and facilitate myosin binding. The greater capacity of C0C2 to activate the thin filament and thus enhance Ca^2+^-sensitivity would help maintain actomyosin interactions. Longer actomyosin interactions would explain why relaxation is prolonged in cardiomyocytes expressing cMyBP-C, as observed in our test of unloaded myocyte shortening. Thin filament activation by cMyBP-C is the strongest of the three isoforms and would prevent dissociation of myosin from actin, despite the decrease in intracellular Ca^2+^. While our studies were not conducted in disease models, unregulated increases in Ca^2+^-sensitivity result in incomplete relaxation and diastolic dysfunction, which is a common phenotype in HCM^37^, which is reported to be caused by mutations in cMyBP-C. An alternate possible explanation for the observed relaxation regulation by cMyBP-C lies in its C0 domain. In our studies, both fsMyBP-C and cMyBP-C similarly regulate contraction at high Ca^2+^ (Fig. 1), suggesting that the C0 domain may not be necessary for regulating contraction, but rather may primarily function as a regulator of relaxation.

Some limitations of our study should be noted. We have focused on the functional effects of the N-terminal region of slow-skeletal, fast-skeletal, and cardiac MyBP-C in the context of cardiac muscle contraction. The use of N-terminal fragments in our experiments enables direct comparison with research in the field^13,23,38^, but MyBP-C N-termini do not exist in isolation. The N-terminal region is part of a much larger protein; the C-terminus of which is responsible for anchoring MyBP-C within the C-zone of the sarcomere^39,40^. Therefore, recombinant MyBP-C proteins may elicit effects outside the C-zone, potentially exaggerating the functional effects of MyBP-C. Furthermore, since the predominant myosin isoforms in human hearts are different from those of mouse, comparisons with, or conclusions drawn from, previous studies will be correspondingly affected. Importantly, our focus on MyBP-C and the thin filament does not preclude the effect of MyBP-C on myosin, and further studies may elucidate how these myofilament proteins affect one another.

In conclusion, our comprehensive experimental approach highlights the nuanced differences between MyBP-C isoforms in the context of regulating the contraction of striated muscles. Specifically, cMyBP-C regulation extends over the full range of Ca^2+^ in contrast to the slow- and fast-skeletal isoforms, which regulate contraction at lower and higher ranges of Ca^2+^, respectively. We postulate that Ca^2+^-range specificity enables cardiac MyBP-C to function over a wide range of Ca^2+^ on a beat-to-beat basis. In contrast, different skeletal muscles contract and relax with variable frequency. Thus, various slow-twitch and fast-twitch skeletal muscles may be fine-tuned for specific functions by expressing different amounts of slow- and fast-skeletal MyBP-C. In contrast, the function of cMyBP-C is highly tunable via post-translational modification^19^ which modulates its interactions with actin and myosin. Phosphorylation sites on ssMyBP-C suggest that skeletal isoforms may be similarly regulated^21^, which will be addressed in future studies focusing on the skeletal muscle system.

## Experimental Methods

### Expression and Purification of Recombinant MyBP-C proteins

Recombinant proteins representing the N-terminal domains up to the C2 domain of slow-skeletal, fast-skeletal, and cardiac isoforms (ssC1C2, fsC1C2, and C0C2, respectively) were generated, purified, and analyzed with SDS-PAGE^41^ (Fig. 1). Each MyBP-C isoform was expressed using a pET28 expression system (Millipore 70777) in *E*. *coli* and included an N-terminal His-tag. Protein expression in BL21 was induced by IPTG and purified through a nickel-nitrilotriacetic acid affinity column in which eluted fractions were combined and dialyzed in PBS. Recombinant proteins were used in all following steady-state biophysical studies to determine the functional differences among the slow-skeletal, fast-skeletal, and cardiac isoforms of MyBP-C.

### Muscle Fiber Bundle Preparation

All experiments in the present study were performed in accordance with the National Institutes of Health Guidelines for the Care and Use of Animals for Experimental Procedures. The experimental protocols were approved by the Institutional Animal Care and Use Committee protocol at Loyola University Chicago, Maywood, IL. In a sterile environment, male Sprague-Dawley rats (250 g, Harlan) were euthanized using Beuthanasia-D solution, and the whole heart was rapidly excised and submitted to retrograde perfusion with Krebs-Henseleit Buffer (KHB) via proximal aorta. The heart was cut to carefully expose the papillary muscles in the left ventricle. Papillary muscles were carefully excised under a dissecting scope (Zeiss Discover.V8 Stereo, PlanAPO S 0.63 × FWD 81 mm) and permeabilized overnight in 1% Triton X-100 (Sigma) in mounting relaxing solution (6.3 mM ATP, 6.48 mM Mg Cl_2_, 10 mM EGTA, 10 mM Na_2_CrP, 49.76 mM Kprop, 100 mM BES, pH 7) at 4 °C, removing cell membrane and membrane-bound proteins. After overnight permeabilization, papillary muscles were further trimmed into fiber bundles approximately 1 mm in length under the dissecting microscope. Straight and parallel fiber bundles were selected based on uniformity and attached at each end with aluminum t-clips. Images were taken under the dissecting scope using an attached digital camera (Canon EOS Rebel T3i). Each fiber bundle was gently cleansed by transferring between a series of 4 washes containing fresh relaxing solution on ice and used within 12 hours. The t-clipped fibers were attached to a force transducer (BAM21-LC; World Precision Instruments, Sarasota, FL) and high-speed length controller (Aurora Scientific, Inc.). Muscle dimensions (cross-sectional area, length, and volume) were determined using an ocular micrometer mounted in the dissection microscope (resolution, ~10 μm). These muscle dimensions were used to normalize contractile force, sarcomere length (SL) and ATPase activity. SL was measured in passive relaxed condition by laser diffraction as previously described^42^, adjusted to, and maintained at 2.0 μm. Briefly, a helium-neon laser beam was directed through the isolated muscle fiber bundle, in which muscle striation pattern diffracts the laser beam into a diffraction pattern. The striations of the muscle fiber bundle act as a grating, with known angles of diffraction, and the projected pattern can therefore be used to measure average SL.

### Incubating with MyBP-C N-termini

The permeabilized fibers were incubated with 10 μM of the ssC1C2, fsC1C2, and C0C2 recombinant proteins or unincubated for 3 minutes in relaxing solution, followed by 3 min in preactivating solution, before measuring force in activating solution. Activating solution contained 20 mM Ca^2+^-EGTA, 1.55 mM potassium propionate, 6.59 mM MgCl_2_, 100 mM N,N-bis (2-hydroxyethyl) taurine; N,N-Bis-(2-hydroxyethyl)-2-aminoethanesulfonic acid, 5 mM sodium azide, 1 mM DTT, 10 mM phosphoenolpyruvate, 0.01 mM oligomycin, 0.1 mM PMSF, 0.02 mM A2P5, and a commercial protease inhibitor cocktail (Sigma). Relaxing solution was the same as activating solution, but contained 20 mM EGTA, 21.2 mM potassium propionate, 7.11 mM MgCl_2_, and no calcium. Preactivating solution was the same as the activating solution, but contained 0.5 mM EGTA, 19.5 mM 1,6-diaminohexane-N,N,N,N’-tetraacetic acid, 21.8 mM potassium propionate, and no calcium. All solutions contained 0.5 mg/ml pyruvate kinase and 0.05 mg/ml lactate dehydrogenase (Sigma) and had an ionic strength of 180 mM, 5 mM MgATP, and 1 mM free magnesium. Recombinant proteins were present in relaxing and preactivation solutions for subsequent measurements. The activation solution did not have recombinant proteins present to avoid potential complications with changes in Ca^2+^ levels. Isometric tension and ATPase activity were measured at various levels of Ca^2+^ activation as previously described^43^. Briefly, the isolated muscle was exposed to a range of calcium solutions obtained by proportional mixing of activating and relaxing solutions. The force generated and ATP consumed were measured simultaneously during the contraction.

### Force-pCa, Force-ATPase, and rate of tension redevelopment

Force-pCa relationship was determined by titrating the activation and relaxing solutions^44^. Maximal isometric tension was measured at 100% activation solution, followed by sequential washes in relaxing and preactivating solutions for 5 minutes each. However, subsequent measurements of isometric tension occurred in solutions titrated with decreasing ratio of activation-to-relaxation solutions, which corresponded to pCa values of 4.50, 5.11, 5.42, 5.61, 5.77, 6.00, and 10.00, respectively, as determined by the Fabiato program^45^. The integrity of the fibers was tested afterwards by measuring maximal tension after the experiment. Any fibers that did not maintain 80%, or greater, maximal tension were excluded from analysis. Force-ATPase relationship was determined using an optical absorbance enzyme assay^44^. Briefly, ATP consumption was determined by measuring the absorbance of UV light at a wavelength of 340 nm. The hydrolysis ATP to ADP and inorganic phosphate is stoichiometrically coupled to the oxidation of NADH to NAD+. This oxidization reaction was catalyzed by pyruvate kinase and lactate dehydrogenase in our activating solutions. NADH, but not NAD+, absorbs light specifically at 340 nm. A series of 50 nl of 10 mM ADP was injected into the measuring chamber. Each injection of ADP induced a rapid reduction in fluorescence and allowed the calculation of the rate of ATP consumption, as determined by measuring the fluorescent decay rate at 340 nm. After completion of the Force-ATPase assay, muscles underwent a rapid release-restretch maneuver in order to break previously formed cross-bridges and allow cross-bridges to reform. The rate constant of tension redevelopment (*k*_tr_) was then measured at maximal activation, as previously described^46^. Force-pCa and Force-ATPase relationships were fitted with a modified Hill equation. Stiffness and tension costs were fitted linearly to the Force-stiffness and Force-ATPase data, respectively.

### *In vitro* Motility Assay

For *in vitro* motility assays, actin filament movements over a cardiac myosin-coated flow cell were observed by epifluorescence microscopy^13^. The experimental flow cell was maintained at 22 °C. Briefly, cardiac myosin from mice (100 µg/mL) were incubated in a nitrocellulose-coated flow cell (2 minutes), followed by blocking with two aliquots of BSA (1 mg/mL) in actin buffer (AB: 25 mM KCl, 1 mM EGTA, 10 mM DTT, 25 mM imidazole, 4 mM MgCl_2_, pH 7.4). One μΜ of unlabeled native thin filaments (NTFs) in AB was added to the flow cell for 1 minute. To eliminate myosin-bound actin, the flow cell was washed twice with 1 mM ATP in AB, followed by two washes with AB alone. Two aliquots of tetramethyl-rhodamine-phalloidin-labeled NTFs were added to the flow cell for 1 minute and rinsed three times with just AB. Finally, to observe NTF movement, we added motility buffer (MB: actin buffer with 100 μΜ ATP, 0.5% methyl cellulose, and CaCl_2_) to the flow cell. The motility buffer contained a range of calcium concentrations, presented as pCa (−log [Ca^2+^]), and ranged from pCa 4–9, as determined using MaxChelator software^47^. We used a Lumen 200 W metal arc lamp (Prior Scientific), Nikon Eclipse TiU microscope with Plan Apo objective (100×, 1.35 n.a.) and Mega Z 10 bit digital camera (Stanford Photonics) for fluorescent excitation of labeled NTFs and image acquisition, respectively. Images were acquired using Piper software at 10 frame/s without pixel binning (95 nm/pixel) and down-sampled to 2 frames/s using ImageJ 1.43 u (NIH). We analyzed the images for velocity of individual NTFs and percentage of mobile NTFs in each movie using DiaTrack 3.03 software (Semasopht). Data were collected as the velocity of moving and percentage of moving filaments from individual movies (Supplemental Figure S2). Data are presented as mean velocity × fraction of moving NTFs ± SEM from triplicate independent experiments (Fig. 2). These data were plotted against pCa and fitted to a sigmoidal dose-response curve, with pCa_50_ representing changes in calcium sensitivity.

### Electron microscopy

For control thin filaments in low Ca^2+^ condition, F-actin, tropomyosin, and troponin were mixed at a molar ratio of 7:2:2 in buffer^48^ (100 mM KAcetate, 2 mM MgCl_2_, 0.2 mM EGTA, 1 mM DTT, 10 mM MOPS, pH 7.0), as reported in previous thin filament studies^13,16,49^. The two-fold molar excess of Tm and Tn was used to maintain binding of these regulatory proteins at the low protein concentrations needed for EM. These reconstituted thin filaments and F-actin were then decorated with the recombinant N-terminal MyBP-C proteins. Thin filaments at a concentration of 2 µM actin were mixed with 6 µM ssC1C2, fsC1C2, or C0C2 (i.e., a 1:3 ratio of actin subunits: C1C2 or C0C2) under the same buffer conditions. After mixing, solutions were incubated at room temperature for 30 min. Five µl aliquots were then applied to EM grids coated with thin carbon supported by a holey carbon film and then negatively stained with 1% uranyl acetate^50^. Dried grids were observed in a Philips CM120 electron microscope (FEI, Hillsboro, OR) at 80 KV under low-dose conditions. Images of filaments were acquired at a pixel size of 0.35 nm, using a 2 K x 2 K CCD camera (F224HD, TVIPS GmbH, Gauting, Germany).

### 3D Reconstruction

Long and relatively straight filaments were selected for 3D reconstruction and straightened with ImageJ (NIH), as previously described^13^. Straightened filaments were converted to SPIDER format (EM2EM; Image Science and Imperial College, London, UK) and cut into segments in SPIDER (v11.2, Wadsworth Center, Albany, NY). Iterative Helical Real-Space Reconstruction (IHRSR) was carried out in SPIDER^51^, using an F-actin model as an initial reference. The process was repeated for 20 rounds, with convergence usually occurring within 10 rounds. UCSF Chimera^52^ was used for visualization, analysis, and atomic fitting of 3D volumes.

### Comparison and Atomic Fitting of Reconstructions

Accurate matching of reconstructions to each other was essential to determine whether MyBP-C N-termini resulted in tropomyosin movement. This was achieved using ChimeraX, as described previously^13^. Reconstructions were fitted with atomic models of F-actin or F-actin-tropomyosin using the ChimeraX “fit in map” tool^13^.

### Computational modeling

We used a computational model that had previously been demonstrated to predict changes in unloaded shortening of individual cardiomyocytes^53^. The myofilament model accounts for length-dependent activation and strain-dependent XB kinetics^54,55^. Since our goal was to model unloaded shortening, in which individual cardiomyocytes are isolated, components accounting for force^55,56^ were set to zero. Computer modelling accounts for differential capacity of MyBP-C to activate the thin filament, defined as k_on_, or the rate of tropomyosin shift from blocked to closed position (see Supplemental Table S4).

### Isolation of adult rat cardiomyocytes

Cardiomyocytes from adult Sprague-Dawley rats (250 g) were isolated as previously described^27^. After isolation, cardiomyocytes were gradually switched into plating media, which contained HMEM supplemented with 100 U/mL penicillin/streptomycin (pen/strep), 10% fetal bovine serum, and 10 mM BDM. For functional measurements, cardiomyocytes were plated on laminin-coated 25 mm coverslips. For protein analysis, cells were directly plated onto laminin-coated 35 mm circular dishes. After 1-hour incubation, plating media were removed, and culture media were added. Culture media consisted of HMEM, supplemented with 0.1% BSA, 100UmL pen/strep, 2 mM glucose, 10 mM BDM, and 5ug/mL insulin/transferrin/selenium (ITS) supplement. Cells were kept in a 2% CO_2_ incubator to maintain pH of 7.0 and maintained for 48 hours.

### Adenovirus Infection and Cell Culture

Commercially purchased slow- and fast-skeletal MyBP-C clones from Origene (NM_175418.3 and NM_004533, Rockville, MD) were used to construct the adenoviruses overexpressing skeletal isoforms of MyBP-C. cMyBP-C adenoviral vectors were generated by the core facilities at Loyola University Chicago. Adenoviral constructs were created by cotransfection of MyBP-C cDNA into DH5α *E*. *coli*. Adenoviruses containing slow, fast, or cardiac MyBP-C included a CMV promoter and c-Myc tag to distinguish between endogenous and adenovirus-mediated MyBP-C protein expression. Uninfected cells and c-Myc-tagged cMyBP-C-overexpressing cells were used as experimental controls. Adenoviral constructs overexpressing slow, fast, and cardiac MyBP-C (Multiplicity of Infection MOI: 1000) were added to the culture media 1 hour after incubation and allowed to infect cardiomyocytes for 24 hours. Culture media were changed after 24 hours, removing the adenovirus and waste and replenishing nutrients in the media.

### Immunofluorescence Imaging

Cultured adult rat cardiomyocytes were plated on chamber slides (Lab-Tek II, Thermo Scientific) for immunofluorescence imaging. After 48 hours in culture, cells were rinsed twice with PBS and fixed with cold (4 °C) 4% paraformaldehyde for 3 minutes, followed by 1 minute in ice cold (−20 °C) methanol. Cells were then permeabilized in 0.5% Triton X-100 in PBS (20 minutes), 0.1% Triton X-100 twice (10 minutes), and antigen-retrieval solution (0.1 M glycine, pH 7.4) (30 minutes), followed by rinsing three times with PBS. Cells were blocked with 0.1% BSA, 0.1% gelatin, 0.1% Tween-20, and 0.0001% NaN_3_, followed by incubation with primary antibodies for ssMyBP-C (ProSci 6679), fsMyBP-C (ProSci 5651), rabbit c-Myc (1:500 Roche) and mouse α-actinin (1:500 Sigma) overnight. After rinsing with PBS, corresponding secondary antibodies (AlexaFluor rabbit 488 at 1:50 dilution and mouse 568 at 1:50 dilution) were added for 1 hour at room temperature, rinsed, and coverslipped with VectaShield mounting medium (Vector Labs H-1500 10mUL 1.5ug/mL) for imaging with confocal microscopy. Images were taken using a Leica TCS SP5 and processed using ImageJ (NIH).

### Western blot analyses

Detection of endogenous and *de novo* MyBP-C protein in cardiomyocytes was determined by immunoblotting. Adenovirus-infected cardiomyocytes were rinsed with sterile PBS to remove culture media, followed by the addition of 60 uL of urea buffer. Cardiomyocytes were scraped off using a cell lifter (Fisher Scientific) and pipetted into 0.6 mL tubes. Twenty-five uL of 4 × loading dye (0.4% bromophenol blue, 10% ß-mercaptoethanol) were then added. Samples were heated at 100 °C for 5 minutes, separated by gel electrophoresis in 10% SDS-PAGE gels, and blotted onto nitrocellulose membrane at 300 mA for 3 hours. Detection of protein was determined by using antibodies for c-Myc, ssMyBP-C, fsMyBP-C, and cMyBP-C (Sigma C3956, ProSci 6679, ProSci 5651, and Santa Cruz sc-137180, respectively)^6^ (Supplemental Figure S4). Quantification of replacement levels was determined by densitometry analysis normalized to ß-actin loading controls, using ImageJ (NIH). Replacement levels were calculated by signal of cMyc/(cMyc + cMyBP-C), as previously described^57^.

### Unloaded shortening in cultured adult rat ventricular cardiomyocytes

After 48 hours in culture, HMEM media were incrementally replaced with Tyrode’s solution (135 mM NaCl, 4 mM KCl, 1 mM CaCl_2_, 1 mM MgCl_2_, 10 mM d-glucose, and 10 mM HEPES) to limit damage to the cells. Coverslips with attached cardiomyocytes were transferred to a custom stimulation chamber containing platinum electrodes. The chamber was then fitted onto a Nikon microscope stage and stimulated (2.0 ms pulse, 1 Hz, and 20 V). Sarcomere length and SL shortening were measured by a video-based detection system (Ionoptix, Milton, MA). Analysis of contractile function was determined from an average of 10 contractions of individual cardiomyocytes, with 10–20 cardiomyocytes measured per coverslip preparation.

### Statistical Analysis

All data are presented as means ± SEM. Data were analyzed using one-way ANOVA with a Bonferroni post-test. Statistical significance was set at p < 0.05.

## Electronic supplementary material

Figures S1 – S4 and Tables S1 – S4
