# Supplementary material for: Skeletal myosin binding protein-C isoforms regulate thin filament activity in a Ca2+-dependent manner
Source: Sci Rep. 2018 Feb 8;8:2604. doi: 10.1038/s41598-018-21053-1 (PMC5805719; doi:10.1038/s41598-018-21053-1)

**SUPPLEMENTAL DATA**

**Supplemental Figures S1 – S4**

**Supplemental Tables S1 – S4**

**Skeletal myosin binding protein-C isoforms regulate thin filament activity in a Ca2+-dependent manner**

**Brian Leei Lina1, Amy Lib,c1, Ji Young Mund,f1, Michael J. Previsb, Samantha B. Previsb,Stuart G. Campbelle, Cristobal G. dos Remediosc, Pieter de P. Tombea, Roger Craigd, David M. Warshawb and Sakthivel Sadayappana,***

*aDepartment of Cell and Molecular Physiology, Health Sciences Division, Loyola University Chicago, Maywood, IL 60153, USA; bDepartment of Molecular Physiology and Biophysics, University of Vermont, Burlington, VT 05405, USA; cBosch Institute, Discipline of Anatomy and Histology, University of Sydney, Sydney 2006, Australia; dDepartment of Cell and Developmental Biology, University of Massachusetts Medical School, Worcester, MA 01655, USA; eDepartment of Biochemistry, Molecular Biology, and Biophysics, University of Minnesota, Minneapolis, MN 55455, USA; fDepartments of Biomedical Engineering and Cellular and Molecular Physiology, Yale University, New Haven, CT 06520, USA; *Department of Biomedical Laboratory Science, College of Health Sciences, Eulji University, Gyeonggi-Do, Seongnam, Republic of South Korea; **Department of Cellular and Molecular Medicine, University of Arizona, Tucson, AZ 85724*

Running Title: Myosin binding protein-C isoforms and muscle function *in vitro*

1Equally contributed

To whom correspondence should be addressed: Sakthivel Sadayappan, PhD, MBA, Department of Internal Medicine, Heart, Lung and Vascular Institute, Division of Cardiovascular Health and Sciences, College of Medicine, University of Cincinnati, 231 Albert Sabin Way, Cincinnati, OH 45267-0575, USA. Phone: +1 513-558-7498; Fax: +1 513-558-2884; Email: [sadayasl@ucmail.uc.edu](mailto:sadayasl@ucmail.uc.edu)

**Keywords:** muscle activation  muscle regulation  myosin binding protein-C  skeletal isoforms  structure-function


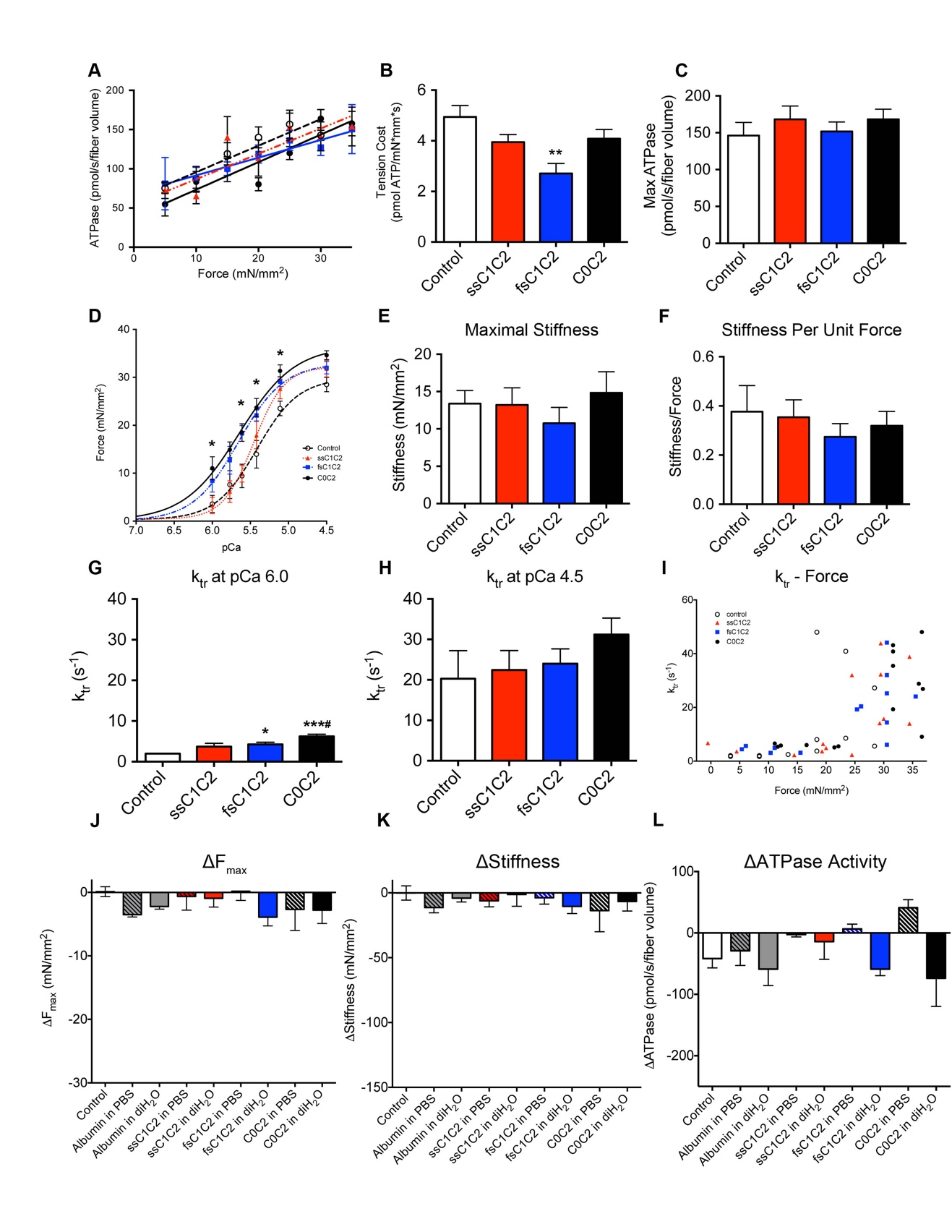
**FIGURE S1**

**Fig. S1.** ATPase activity of fibers shown in Fig. 1 was simultaneously measured. (*A*) ATPase-force relationship demonstrates that fsC1C2 affects myosin ATPase activity. (*B*) Quantification of tension cost, as determined from the slope of the ATPase-Force relationship, demonstrates that fsC1C2 also significantly reduces tension cost. (*C*) Maximal ATPase activity was also unaltered by MyBP-C N-termini. (*D*) fsC1C2 and C0C2 significantly promoted force generation at submaximal, but not maximal, Ca2+ (pCa 4.5) levels, as determined by force-ATPase assay. (*E*) Maximal stiffness was not altered between groups. (*F*) Stiffness-force relationship also demonstrates no significant differences in number of cross-bridges formed per force generated. (*G*) Rate of tension redevelopment (*k*tr) at submaximal activation levels (pCa 6) demonstrated that fsC1C2 and C0C2 promoted *k*tr. (*H*) *k*tr at maximal activation levels (pCa 4.5) was not significantly different between groups. (*I*) *k*­tr-force relationship demonstrates no difference in the correlation between groups. (*J-L*) Neither changes in ionic strength nor protein incubation with chicken egg albumin (a protein of molecular weight similar to that of MyBP-C N-terminal fragments) significantly altered parameters, as determined by (*J*) F­­max­, (*K*)stiffness , and (*L*) ATPase activity. Graphs represented as mean ± SEM, *p<0.05 *vs*. controls, **p<0.01 *vs*. controls, #p<0.05 *vs*. ssC1C2, n=7-9 animals).


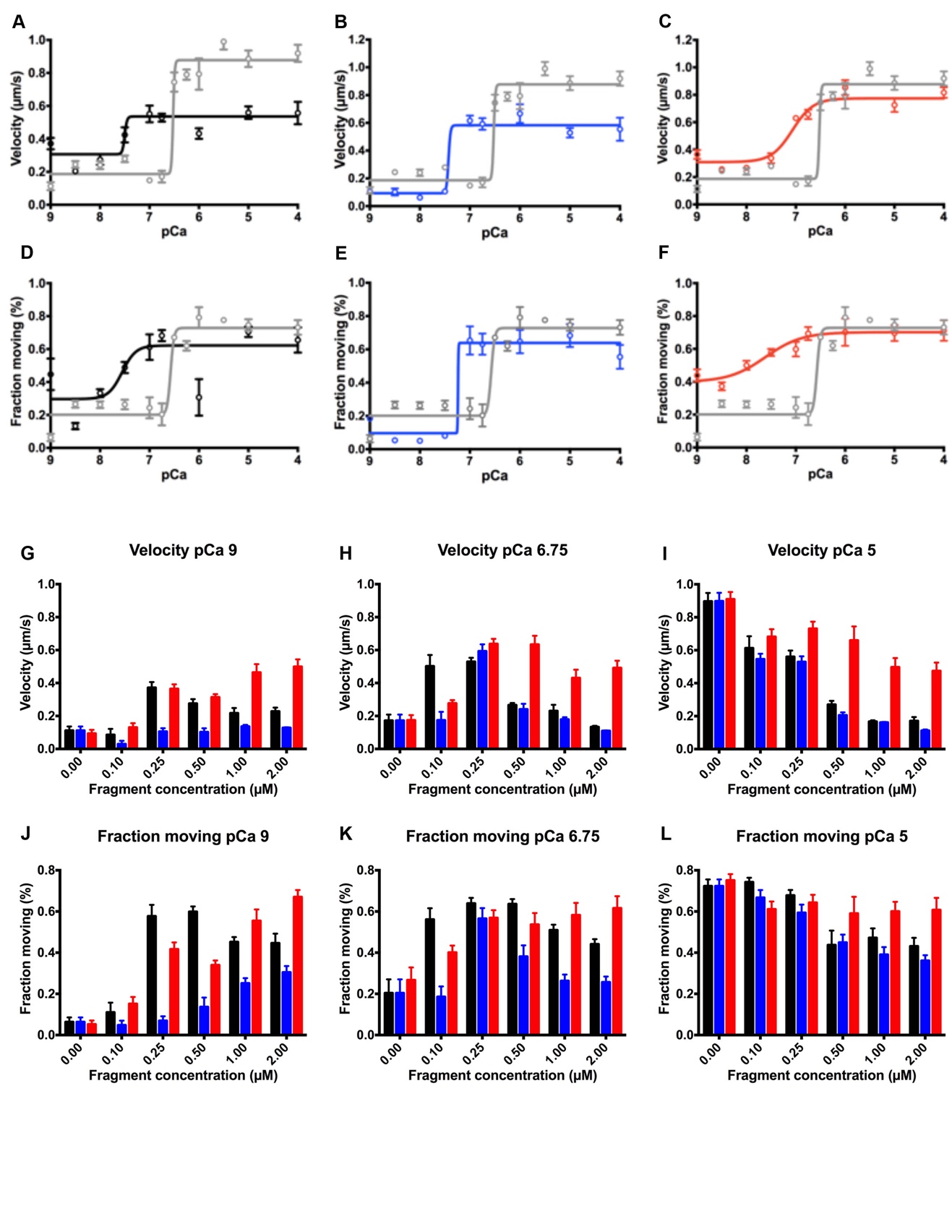
**FIGURE S2**

**Fig. S2.** *In vitro* motility assays demonstrate that ssC1C2 (red), fsC1C2 (blue), and C0C2 (black) regulate contraction by promoting and inhibiting the motility of native thin filament (NTF) at low and high Ca2+, respectively. Motility was calculated by measuring (*A-C*) velocity of NTF sliding (µm/s) and (*D-F*) fraction moving (%). Similarly, concentration-dependent effects of MyBP-C N-termini were determined by (*G-I*) NTF velocity and (*J-L*) fraction moving. NTF motility is often measured as NTF sliding velocity and/or fraction moving. The present study analyzed both individually and together (see Fig. 2).

**FIGURE S3**

**
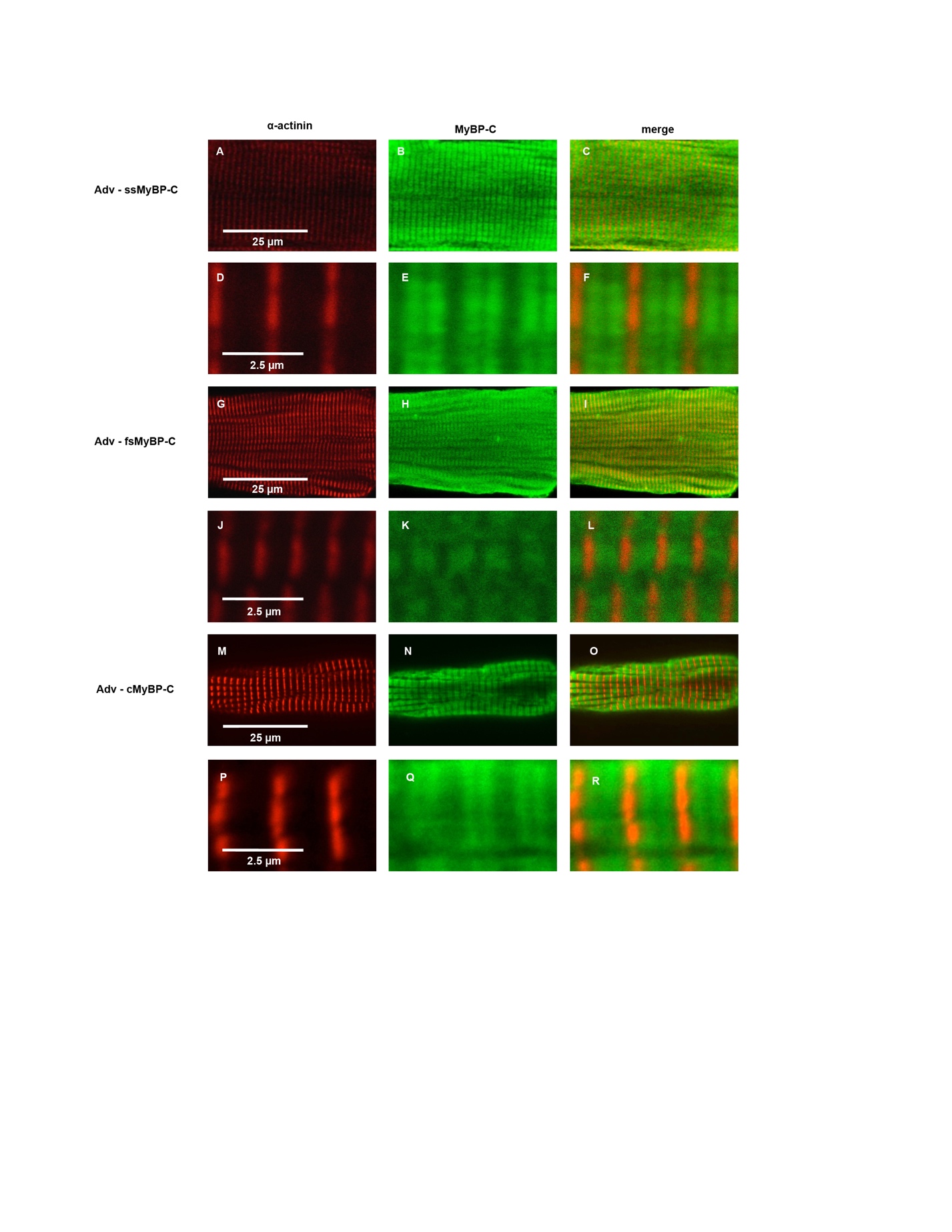
**

**Fig. S3.** ARVMs infected with adenoviral constructs overexpressing full-length, myc-tagged ssMyBP-C, fsMyBP-C and cMyBP-C were cultured for 48 hours. Localization of exogenous (*A-F*) ssMyBP-C, (*G-L*) fsMyBP-C, and (*M-R*) cMyBP-C was detected using immunofluorescence. Staining for α-actinin delineates the location of cardiomyocyte Z-disks, the borders of the sarcomere (*A, D, G, J, M, P*). Localization of adenoviral-mediated overexpressed MyBP-C was detected using antibodies for ssMyBP-C (*B, E*) fsMyBP-C (*H, K*) and cMyc (*N, Q*). Merged images demonstrated that MyBP-C expression did not localize to the Z-disk (*C, F, I, L, O, R*).


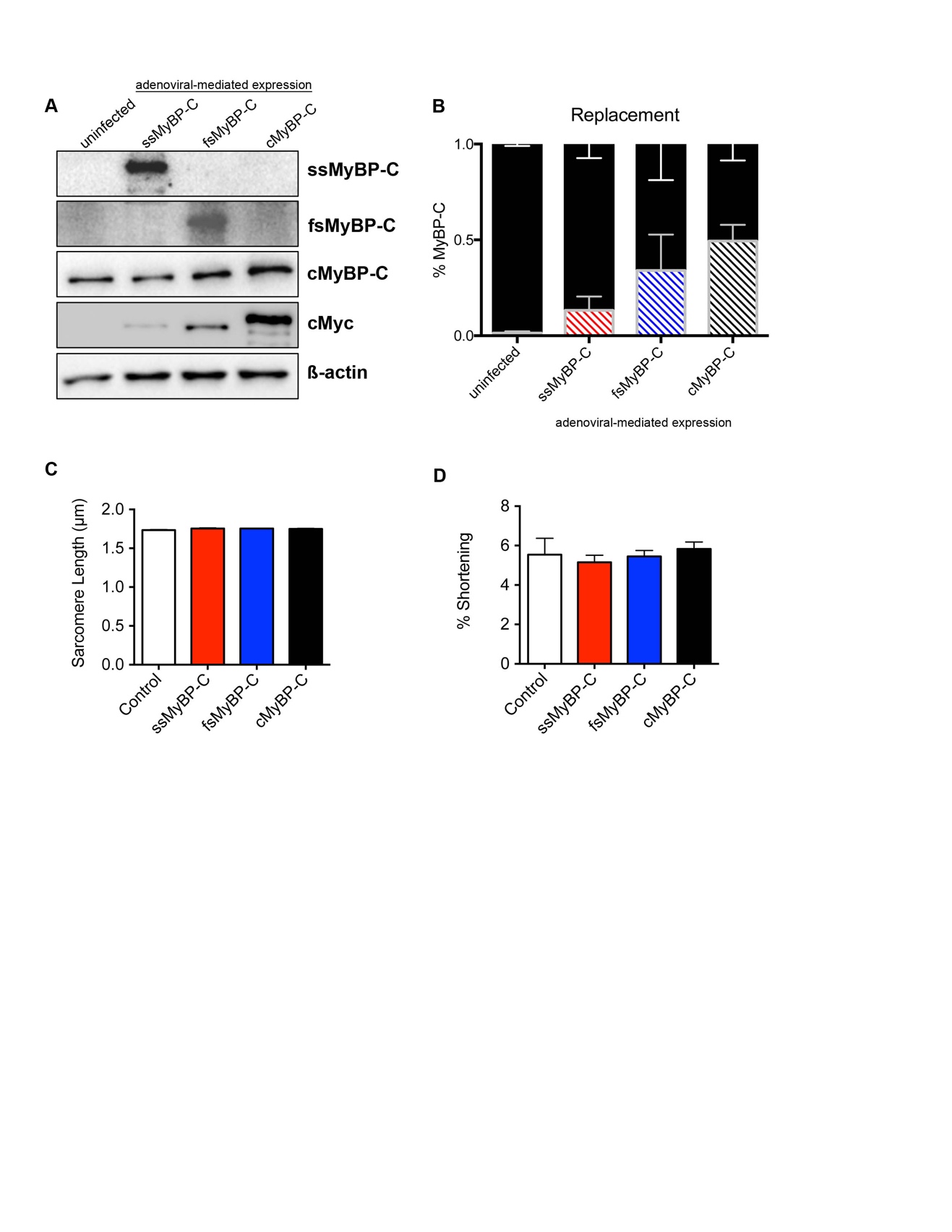
**FIGURE S4**

**Fig. S4.** ARVMs infected with adenoviral constructs overexpressing full-length, myc-tagged MyBP-C isoforms were cultured for 48 hours (MOI 1000). Uninfected ARVMs were included as controls. (*A*) Western blots demonstrate specific expression of ssMyBP-C and fsMyBP-C. Because of the presence of endogenous cMyBP-C in ARVMs, adenoviral-mediated MyBP-C expression was determined by cMyc tag detection. All analyses are normalized to ß-actin. (*B*) Adenoviral-mediated overexpression of ssMyBP-C (red stripes), fsMyBP-C (blue stripes) and cMyBP-C (black stripes) partially replaced endogenous cMyBP-C (black solid), as demonstrated by densitometry analysis. No significant differences were observed in replacement levels among adenoviral-mediated ssMyBP-C, fsMyBP-C, and cMyBP-C. (*C*) Resting sarcomere length of ARVMs was unchanged suggesting similar starting points of contraction. (*D*) Percent shortening also showed no difference among groups, suggesting similar contractility.

**Table S1. N-terminal homology between N-terminal MyBP-C proteins (ssC1C2, fsC1C2, C0C2) and full-length MyBP-C**

| **MyBP-C**  **N-termini** | **ssC1C2**  **(a.a. 1-341)** | **fsC1C2**  **(a.a. 1-337)** | | | **C0C2**  **(a.a. 1-448)** | | |
| --- | --- | --- | --- | --- | --- | --- | --- |
| **NCBI Accession Number** | **NP_780627.2** | **NP_666301.2** | | | **AAI54409.1** | | |
|  |  | **Identity** | **Positives** | **Gaps** | **Identity** | **Positives** | **Gaps** |
| **ssC1C2** |  | 53% | 70% | 5% | 52% | 67% | 6% |
| **fsC1C2** |  |  |  |  | 48% | 59% | 8% |
| **MyBP-C**  **Full-Length** | **ssMyBP-C** | **fsMyBP-C** | | | **cMyBP-C** | | |
|  |  | **Identity** | **Positives** | **Gaps** | **Identity** | **Positives** | **Gaps** |
| **ssMyBP-C** |  | 51% | 70% | 2% | 49% | 67% | 5% |
| **fsMyBP-C** |  |  |  |  | 53% | 68% | 4% |

**Table S2. Effects of MyBP-C N-termini as determined by Force-ATPase assay**

|  | **Control** | **+ssC1C2** | **+fsC1C2** | **+C0C2** |
| --- | --- | --- | --- | --- |
| **pCa50** | 5.2 ± 0.46 | 5.47 ± 0.06 | 5.71 ± 0.06* | 5.6 ± .16* |
| **Tension Cost** | 4.94 ± 0.45 | 3.95 ± 0.30* | 2.7 ± .40** | 4. 1 ± 0.36* |
| **(pmol/s/fiber volume)** |
| ***k*tr (pCa 6)** | 1.97 ± 0.98 | 3.70 ± 1.81 | 4.23 ± 0.50* | 6.22 ± 0.53***# |
| **(s-1)** |
| **Force (pCa 6)** | 0.62 ± 0.32 | 3.27 ± 1.70 | 9.48 ± 2.40** | 9.50 ± 2.09** |
| **(mN/mm2)** |

Values are mean ± SEM. ***p<0.0001, **p<0.01, *p<0.05 vs. control; #p<0.05 vs. ssC1C2 (n=5-9 animals)

**Table S3. Actin and Thin Filament Diameters (nm)**

|  | **Control** | **+ssC1C2** | **+fsC1C2** | **+C0C2** |
| --- | --- | --- | --- | --- |
|  |  |  |  |  |
| **Thin Filament** | 10 ± 0.7 | 16 ± 0.7* | 16 ± 1.1* | 17 ± 1.3* |

Values are mean ± SEM. (*p<0.05 significant change compared to control)

**Table S4. Computational modeling parameters account for differential thin filament activation (kon) capacities of MyBP-C.**

| **MyBP-C isoform** | **kb** | **ku** | **kon** | **koff** | **fapp** | **gapp** | **hf** | **hb** | **gxb** | **gammaB** | **Temp. (K)** |
| --- | --- | --- | --- | --- | --- | --- | --- | --- | --- | --- | --- |
| **ssMyBP-C** | 0.09 | 0.45 | **375** | 0.33 | 0.30 | 70e-3 | 2000e-3 | 400e-3 | 0.25 | 300 | 323 |
| **fsMyBP-C** | 0.09 | 0.45 | **360** | 0.33 | 0.30 | 70e-3 | 2000e-3 | 400e-3 | 0.25 | 300 | 323 |
| **cMyBP-C** | 0.09 | 0.45 | **500** | 0.33 | 0.30 | 70e-3 | 2000e-3 | 400e-3 | 0.25 | 300 | 323 |

kb: rate of Ca2+ binding to troponin C; ku: rate of Ca2+ dissociation from troponin C; kon rate of tropomyosin transition from blocked to closed state; koff: rate of tropomyosin transition from closed to blocked; fapp: rate of XB attachment; gapp: rate of XB detachment from pre-powerstroke state; hf: rate of forward powerstroke; hb: rate of reverse powerstroke; gxb: rate of XB detatchment from the post-powerstroke state; gammaB: cooperative coefficient representing tropomyosin-tropomyosin interactions; Temp: temperature in degrees Kelvin.

**ORIGINAL RAW FILE – WESTERNS**

**Figure 1B**


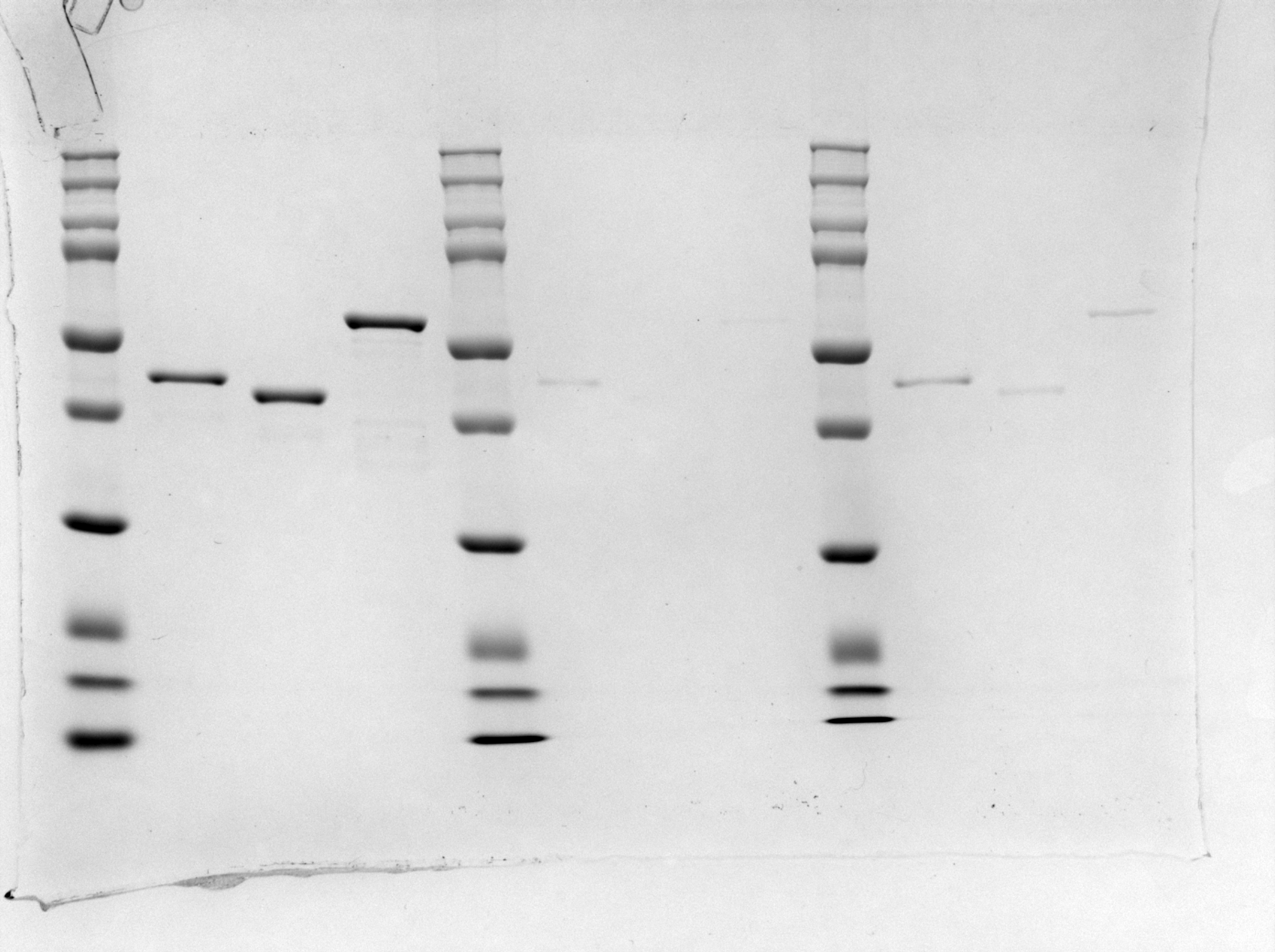


**ORIGINAL RAW FILE – WESTERNS**

**Figure S4 – Panel A – ssMyBP-C**


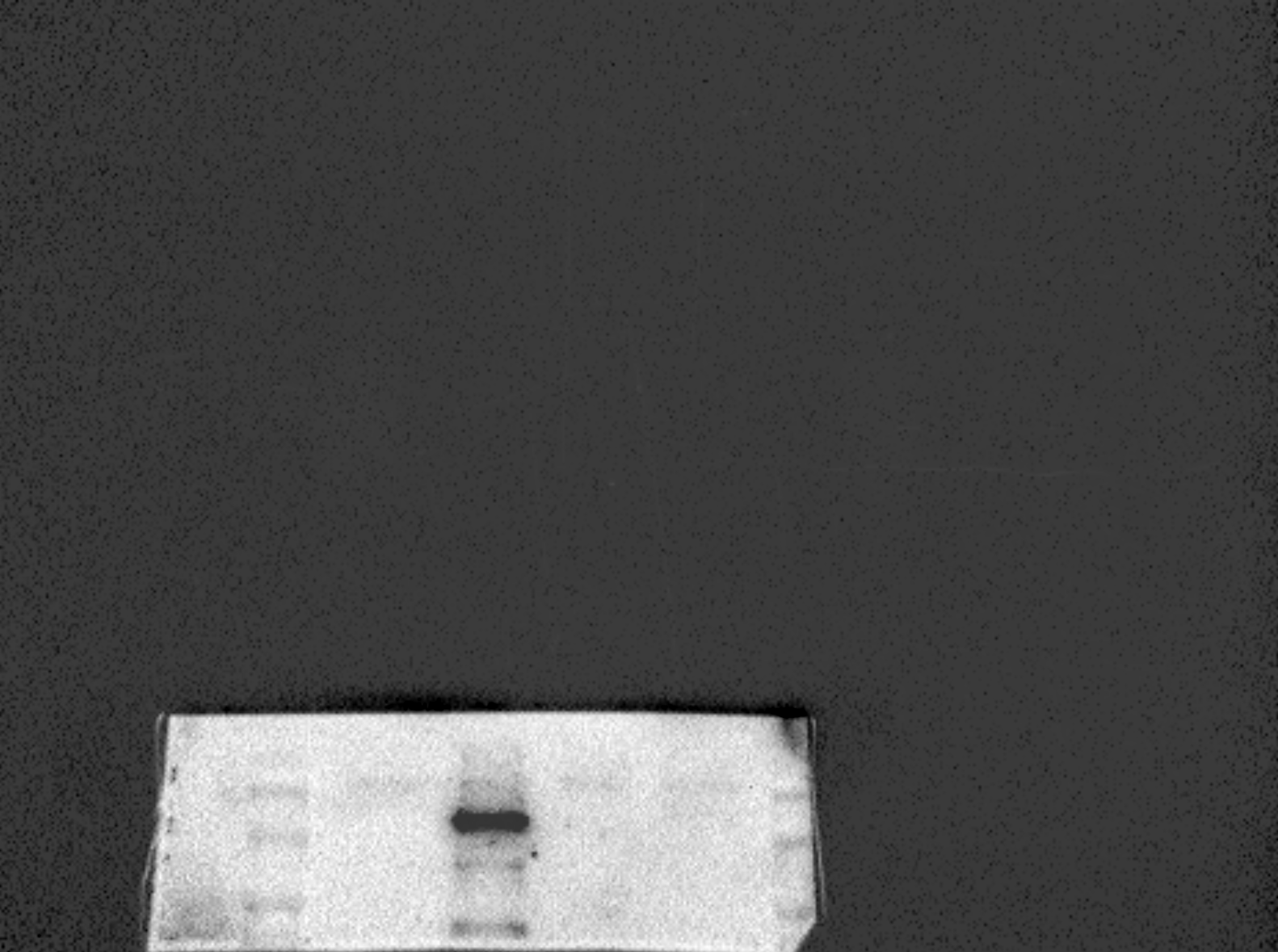


**ORIGINAL RAW FILE – WESTERNS**

**Figure S4 – Panel A – fsMyBP-C**


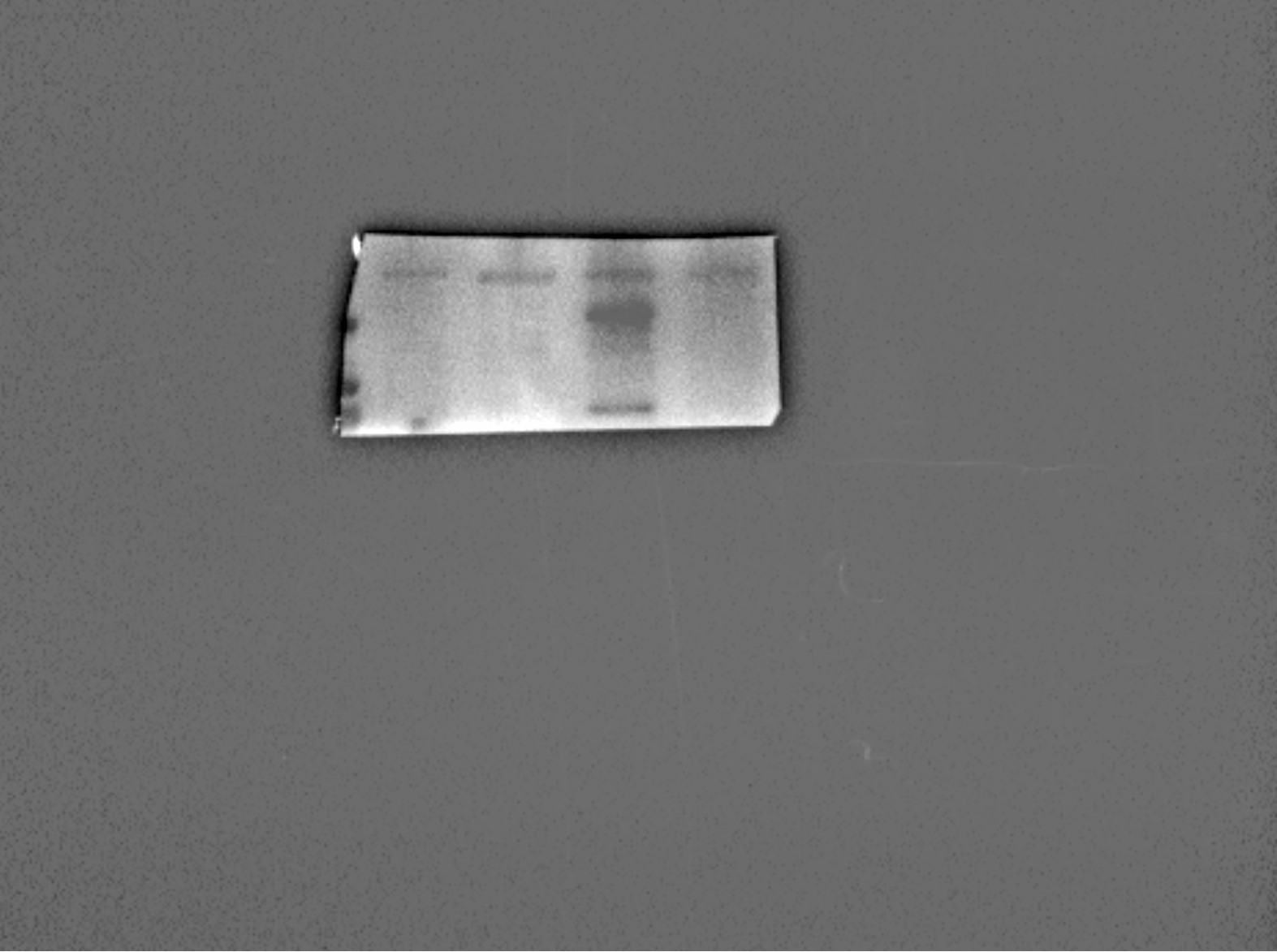


**ORIGINAL RAW FILE – WESTERNS**

**Figure S4 – Panel A – cMyBP-C**


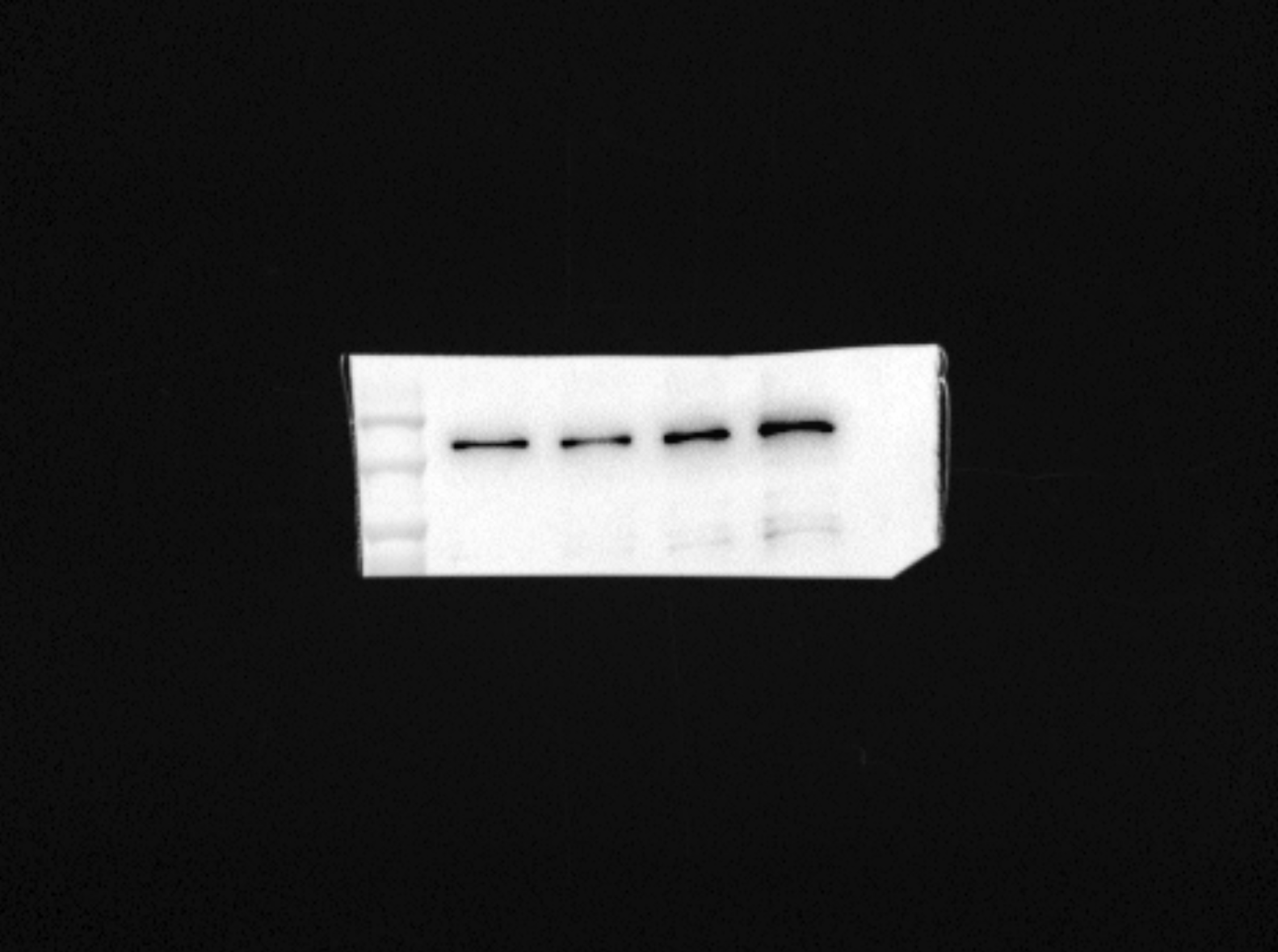


**ORIGINAL RAW FILE – WESTERNS**

**Figure S4 – Panel A – cMyc**


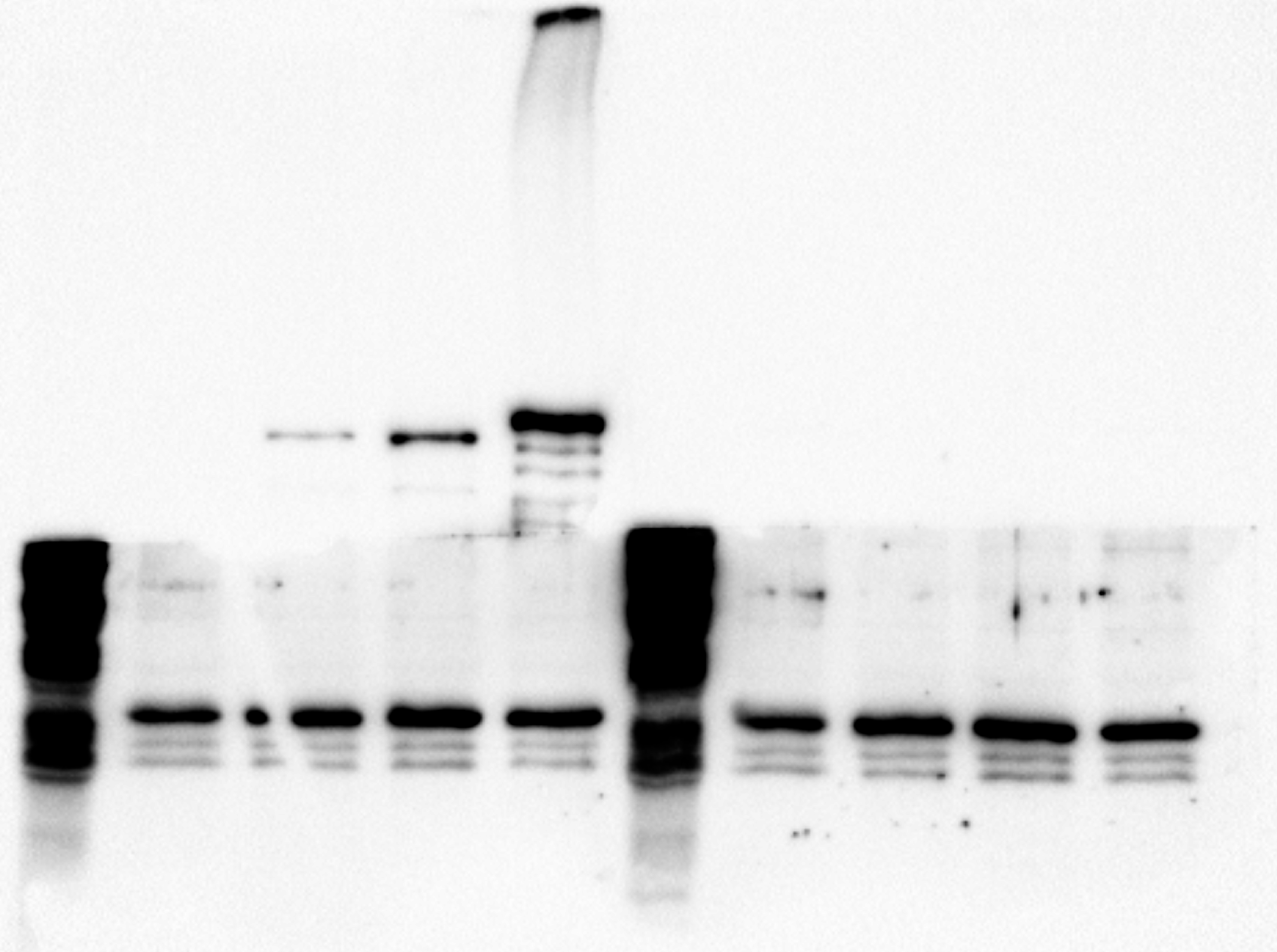


**ORIGINAL RAW FILE – WESTERNS**

**Figure S4 – Panel A – β-actin**


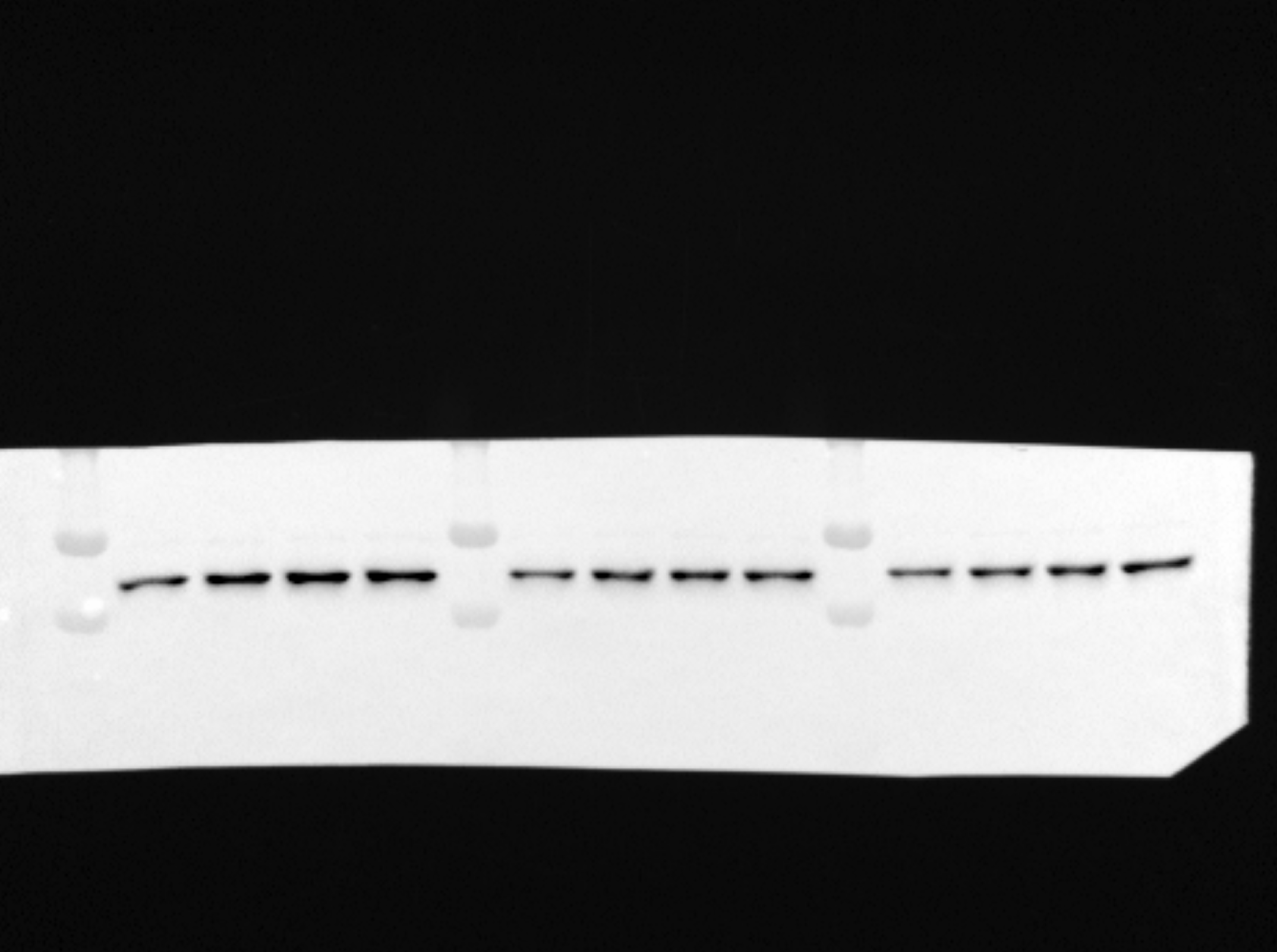

Supplement: Supplementary file 1 — Figures S1 – S4 and Tables S1 – S4 [file 41598_2018_21053_MOESM1_ESM.doc]
